# Antibody drug conjugates (ADCs) charged with HDAC inhibitor for targeted epigenetic modulation†
†Electronic supplementary information (ESI) available: Experimental procedures, biological activity data, NMR spectra for characterisation. See DOI: 10.1039/c7sc05266a


**DOI:** 10.1039/c7sc05266a

**Published:** 2018-07-03

**Authors:** Elena Cini, Valentina Faltoni, Elena Petricci, Maurizio Taddei, Laura Salvini, Giuseppe Giannini, Loredana Vesci, Ferdinando Maria Milazzo, Anna Maria Anastasi, Gianfranco Battistuzzi, Rita De Santis

**Affiliations:** a Dipartimento di Biotecnologie , Chimica e Farmacia , Università degli Studi di Siena , Via A. Moro 2 , 53100 Siena , Italy . Email: maurizio.taddei@unisi.it; b Lead Discovery Siena srl , Via Fiorentina 1 , 53100 Siena , Italy; c Fondazione Toscana Life Sciences , Via Fiorentina 1 , 53100 Siena , Italy; d R&D Alfasigma S.p.A. , Via Pontina, Km. 30.400 , 00071 Pomezia , Roma , Italy . Email: Giuseppe.Giannini@alfasigma.com

## Abstract

We describe here two novel antibody-drug conjugates loaded with the HDAC inhibitor ST7612AA1 (IC_50_ equal to 0.07 μM on NCI-H460 cells), a thiol-based molecule with a moderate toxicity *in vivo*. Two payloads were prepared using cleavable and non-cleavable linkers. After anchoring to cetuximab through amide bond with lysines, the resulting HDAC inhibitor-antibody conjugates showed ability to recognize EGFR and efficient internalization in tumor cells. Both ADCs induced sensible increment of histones 3 and 4 and alpha-tubulin acetylation. Animal models of human solid tumors showed high anti-tumor efficacy of the conjugates without the toxicity generally observed with traditional ADCs delivering highly potent cytotoxic drugs. These compounds, the first ADCs charged with not highly cytotoxic warheads, are potentially suitable for epigenetic modulation, extending the ADC strategy to the targeted delivery of HDAC inhibitors with many possible therapeutic applications beyond cancer.

## Abstract 2

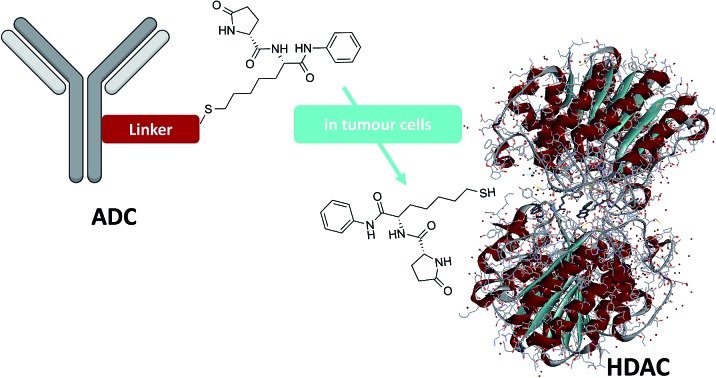
A new weapon is added to the ADC arsenal, a thiol based HDAC inhibitor. Low toxic, hits the target and stops tumor growth in many mouse models.

## Introduction

With more than 50 antibody-drug conjugates (ADC) in clinical trials for treatment of oncological diseases, the ADC approach to chemotherapy is finding renewed interest, especially after Adcetris (FDA, 2011), Kadcyla (FDA, 2013) and more recently Besponsa (FDA, 2017) approval for treatment of CD30 + Hodgkin lymphoma, Her-2-positive metastatic breast cancer and acute lymphoblastic leukemia (ALL), respectively.^1^ While the use of monoclonal antibodies (mAbs) and molecularly diverse linkers has been widely represented, only highly potent cytotoxic drugs like microtubule inhibitors maytansines (DM1/DM4) or auristatins (MMAE/MMAF) dominate the current ADC landscape. Despite a relatively poor clinical success rate, ∼70% of the ADCs currently in clinical trials contains payloads belonging to these classes of molecules.^2^ Other cytotoxic drugs include molecules targeting the DNA minor groove,^3^,^4^ and topoisomerase I inhibitors.^5^ Since only <1% of the injected dose is expected to target the tumor, the presence of payloads active in nano- or picomolar ranges is thought to be a strict requirement for ADC-based therapies.^6^ Consequently, most of the ADC toxicity observed in patients is deriving from off-target effects due to linker instability. ADCs containing microtubule inhibitors induce peripheral neuropathy, neutropenia, gastrointestinal thrombocytopenia,^7^ hepatic and ocular toxicities^8^ while calicheamicin based ADCs cause thrombocytopenia and hepatic dysfunction.^9^ With very few exceptions, until now the paradigm that an efficient ADC must be charged with a highly potent payload has guided ADC research and development. However, the side effects associated with intrinsic cytotoxicity of the payloads are a serious limit to the ADC applicability in therapies beyond cancer.^10^ In addition, the potency of the cytotoxic payload implies manufacturing problems in ADC production, because of containment restrictions required to guarantee protection of operators and environment.^11^ Compared to the progress in development of ADCs in oncology, few ADC candidates have been investigated for the treatment of other diseases. Dexamethasone and budesonide have been linked to anti-CD70 antibodies for anti-inflammatory and immunosuppressive therapies.^12^ The highly cytotoxic Src kinase inhibitor dasatinib (IC_50_ < 1 nM) has been also linked to an antibody to produce an immunosuppressive ADC.^13^ Finally, the recent discovery that a modified rifampicin bonded to an anti-*Streptococcus aureus* antibody eradicates intracellular infection has raised great expectations for fighting bacterial antibiotic resistance with ADCs.^14^

**Scheme 1:**
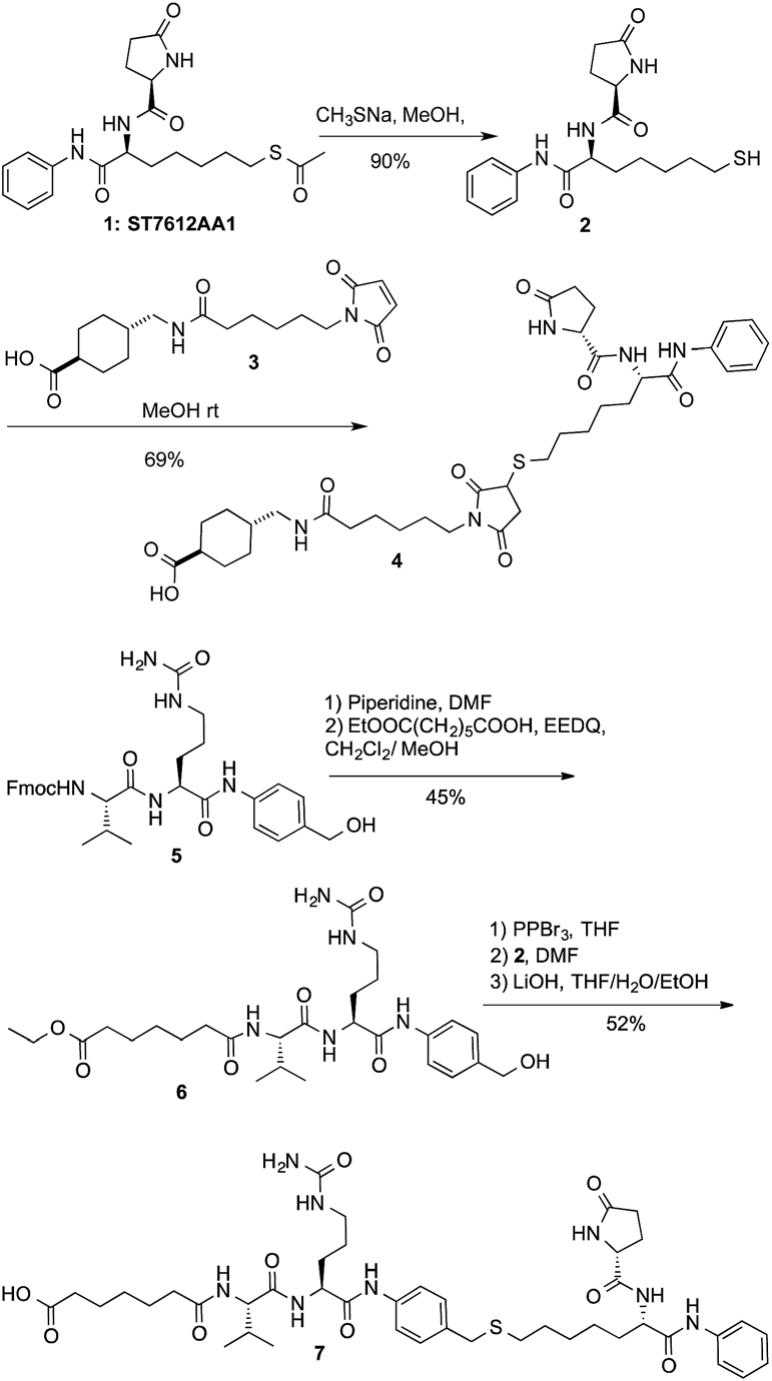
Preparation of payloads/linkers for conjugation.

Epigenetic aberrations contribute to the onset and progression of several diseases *via* the gain or loss of function in epigenetic regulatory proteins.^15^ Deacetylating enzymes are valuable targets to treat aberrant deacetylations occurring in neurological disorders, inflammation, viral and protozoal infections, cardiovascular disorders and cancer.^16^ We recently discovered ST7612AA1 (ref. 17) (**1**, Scheme 1), a new thiol-based histone deacetylase (HDAC) inhibitor that slows down *in vivo* and *in vitro* growth of several tumors such as Ras-mutant colon carcinoma, non-small cell lung tumors, ovarian cancer, triple-negative breast cancer (TNBC), acute myeloid leukemia, and diffuse large B cell lymphoma. Moreover, **1** showed to modulate the NF-κB pathway and epithelial–mesenchymal transition (EMT), as well as transcripts involved in immune response and in key pathogenic pathways, suggesting the potential use in management of inflammatory diseases.^18^ Compound **1** also proved to be active in HIV reactivation, with potential applications in therapies aiming at the eradication of the viral reservoirs.^19^ Compound **1** is the pro-drug of the corresponding thiol **2** (Scheme 1) that is rapidly formed in plasma after the injection. A high affinity of **2** with some histones isoforms (IC_50_ 13, 5, 3 and 11 nM on HDAC-1, -3, -6 and -10 respectively) was observed together with a promising activity of **1** on tumour cell lines (IC_50_ = 0.07 μM on NCI-H460 cells) and a moderate toxicity *in vivo*.^17^

## Results and discussion

Starting from this standpoint, we thought to exploit the features of compound **1** into a new concept of ADC directed towards histone targets. The release of thiol **2** upon internalization of a HDAC charged ADC, may open the way to selectively target different HDAC isoforms with many potential applications in cancer therapy and beyond, and other indications that might possibly have benefit from epigenetic modulations.

The clue of an effective ADC lies in its linker. Generally, linkers are designed in such a way to discharge the drug intracellularly through a controlled process. More stable linkers can release the free toxin by unspecific endosomal degradation,^2^ while Cathepsin B cleavable linkers, discharge the payloads upon lysosomal processing.^20^ As the active form of ST7612AA1 (**1**) is a thiol (**2**), two alternative conjugation strategies were exploited: (i) a linker based on the Michael addition of the thiol to maleimide that might be cleaved by catabolism (sharing features with the linker present in Kadcyla);^21^ (ii) a cleavable linker based on the Val-Cit dipeptide^22^ bonded to a *p*-amino-benzyl (PAB) self-immolative group.^23^,^24^ Both linkers were attached through a stable amide bond with lysines to cetuximab (Ctx), a monoclonal antibody (mAb) specific for the Epidermal Growth Factor Receptor (EGFR). The linker-payloads **4** and **7** were thus prepared as described in Scheme 1. After alkaline deacetylation of **1**, Michael addition of thiol **2** to **3** gave product **4** ready for lysine coupling after *N*-hydroxysuccinimide (NHS) *in situ* activation. Compound **7** was prepared starting from dipeptide **5** after deprotection and coupling with monoethyl pimelate in the presence of EEDQ to give **6**. The hydroxy group was then transformed into the corresponding bromide not stable enough for isolation. However, direct nucleophilic substitution with thiol **2**, followed by ester hydrolysis, gave acid **7** (Scheme 1). Compounds **4** and **7** were stable in PBS or in mouse plasma. However, after 5 h, hydrolysis of the five-membered ring of **4** started giving an almost complete transformation into the monoamide of succinic acid in 12 h, as revealed by NMR and MS/ESI (see ESI†).

This opened succinimide is stable in solution as no trace of compound **2** was observed in the further 72 h.

This increased stability of ring opened succinimides have been already documented in ADCs where maleimide was employed for anchoring payloads to cysteines residues in the mAb.^25^

**Scheme 2:**
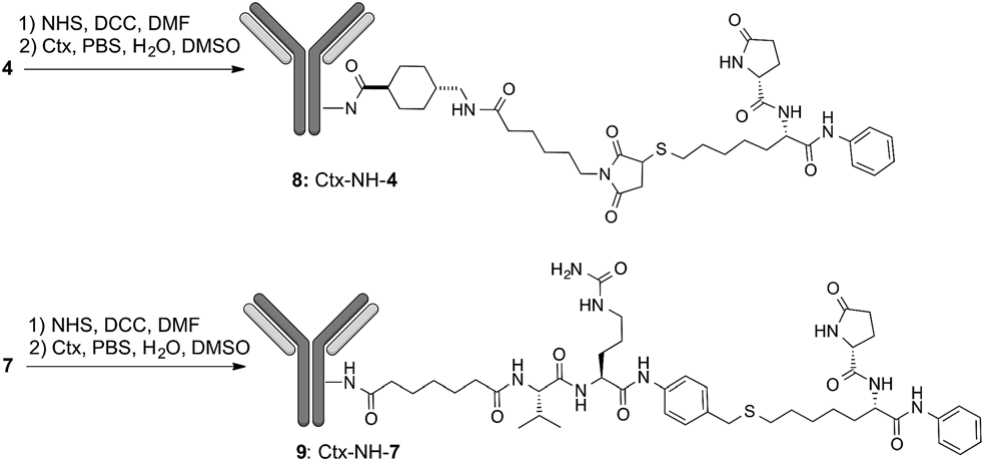
Preparation of ADCs **8**: DAR = 8 (±1), average on 6 batches; and ADC **9**: DAR = 6 (±0.5), average on 3 batches.

Lysine anchoring was carried out with compounds **4** and **7** after activation with NHS and incubation in DMSO/H_2_O/PBS buffer (pH 7.4) with Ctx at room temperature using a 20 molar fold excess of the linker respect to the mAb (Scheme 2).

Purification of **8** and **9** was carried out by dialysis while conjugation and DAR were determined by MALDI analysis that showed DAR = 8(±1) for compound **8** and DAR = 6(±0.5) for compound **9** (Fig. S1 and S2†). As expected, by HIC analysis the conjugates **8** and **9** showed a large distribution of molecular weights with less than 10% of unreacted antibody in any sample. Size exclusion chromatography showed a major peak of the size expected for conjugates **8** and **9** with minor peaks referable to aggregated and degraded forms, respectively (Fig. S3†).

The binding specificity of conjugates **8** and **9** was than confirmed by flow-cytometry (FACS analysis) on Capan-1 (human pancreas carcinoma), NCI-H1975, A549 (human lung carcinoma), and SK-MEL-28 (human melanoma) cell lines, a panel of EGFR positive and negative tumour cells (Fig. S4†). Immunoreactivity was tested by antigen-specific ELISA on a 96-well plate coated with 50 ng per well of recombinant human EGF-R/ErbB1 Fc chimera. Both ADCs showed reactivity with their specific target with potency comparable (ADC **8**) or slightly but significantly lower (ADC **9**) to that of Ctx (Fig. S5a†). Consistently, affinity measurements by surface plasmon resonance (Biacore system) showed an apparent higher affinity of **8** compared to **9** although both derivatives exhibit antigen interaction kinetics in the same sub-nanomolar range of Ctx (Fig. S5b†).

**Fig. 1:**
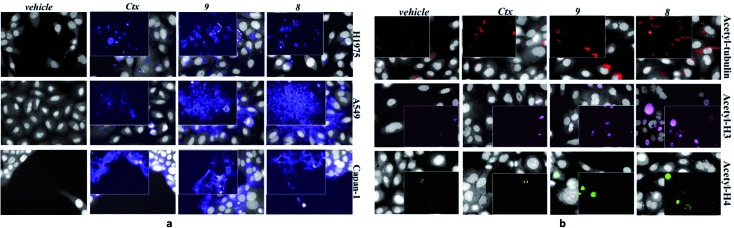
(a) Internalization of Ctx and ADCs **8** and **9** in Capan-1 (pancreas carcinoma), A549 and NCI-H1975 (lung carcinoma) human cells, and (b) their effect on acetylation of HDAC-target proteins in NCI-H1975 cells. Insets show specific fluorescence signals within the cells. Each image is representative of at least 5 fields of duplicate wells. Magnification 60×. Data are from one representative experiment out of two.

After securing that conjugation did not modify the Ctx properties, ADC internalization, an essential step for biological function, was investigated on the EGFR-expressing tumour cell lines Capan-1, NCI-H1975, A549 (Fig. 1a), and on EGFR-negative cells (Fig. S6b†). After cell treatment with Alexa Fluor488-labeled ADCs, fluorescence was observed by High Content Screening (HCS) imaging analysis. Results show that, upon binding to EGFR, ADCs **8** and **9** are internalized in a comparable manner respect to Ctx, resulting in accumulation within the internal vesicles of the multivesicular bodies and subsequent translocation to the lysosomal compartment (Fig. 1a).

**Fig. 2:**
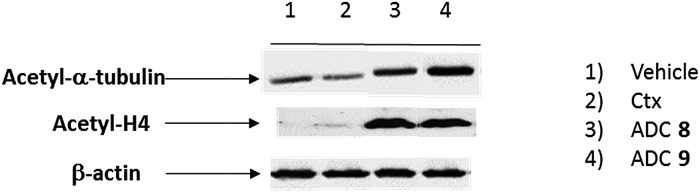
Effect of native Ctx (lane 2), ADCs **8** or **9** (lanes 3 and 4, respectively) on acetylation of alpha-tubulin and histone H4 in A549 (human lung carcinoma) cells. Cells were cultivated 3 hours at 37 °C with medium (lane 1) or antibodies (20 μg mL^–1^) and then western blot analysis was carried out on total protein lysates. Beta-actin was used for normalization. One representative blot is shown.

To demonstrate that the HDAC inhibitor charged ADCs **8** and **9** are suitable for epigenetic modulation, the release of **2** was then proven in NCI-H1975 cells by detecting acetylation of alpha-tubulin and histones, a clear mark of specific inhibition of HDAC6 and nuclear HDAC isoforms,^26^ respectively (Fig. 1b). Fluorescence data showed that all tested ADCs induced a relevant increase in the acetylation level of both alpha-tubulin and histones H3 and H4, as result of direct enzymatic inhibition of HDAC6 and class I HDACs, respectively while no effect was observed in cells treated with Ctx alone (Fig. 1b), in EGFR-negative cells and in other cell lines assessed (Fig. S7 and S8†). Increased acetylation of tubulin and histone H4 was also confirmed in A549 cells by western blot analysis of total protein lysate (Fig. 2). Comparison of band intensity clearly shows an increment of acetylation when cells are treated with ADCs **8** and **9** if compared with the vehicle-treated cells and with cells treated with Ctx.

This result demonstrates the ability of ADCs to be internalized and to acts as a HDAC inhibitor, due to the presence of compound **2** indeed.^27^

Release of **2** from ADC **8** occurred by treatment with human hepatic microsomes.^28^ After 72 h of incubation at 37 °C, quantitative HRMS analysis showed the presence of the peaks at *m*/*z* = 362.1548 and 328.1671 referable exclusively to compound **2** in concentration 1.9 μM (29% of release referred to the amount of payload present in the starting ADC.) When ADC **9** was submitted to the same procedure, with evidence of **2** was found. The linker Val-Cit-PAB conjugated with **2** is known to be cleaved by lysosomal Cathepsin B.

However, in our hands, different incubation experiments carried out on compound **7** or ADC **9** with the isolate enzyme or hepatic microsomes, never gave convincing proofs of the presence of **2** or, at least, of the PAB-thioether derived from **2** by amide cleavage. Cellular metabolism might generate the drug attached to amino acid fragments derived from the antibody^29^–^31^ and, successively, **2**. Submitting the structure **7** to MetaSite, a software that predicts metabolic transformations related to cytochrome and monooxygenase mediated reactions,^32^**2** is revealed as a possible metabolite (ESI†). This putative metabolic mediated release of **2** from ADC **9** may explain why this ADC is only slightly more therapeutically potent than Ctx alone suggesting also that the linkage through Val-Cit PAB was not a good choice for a correct release.

However, the activity of ADC in epigenetic modulation can be explained exclusively assuming that **2** is released. The largely accepted pharmacophore model for most of the known hydroxamic acid inhibitors consists of (a) a capping group that interacts with the residues at the active site entrance (cap), (b) a zinc binding group (ZBG) that coordinates to the catalytic metal atom within the active site, and (c) a linker that binds to the hydrophobic channel and helps Cap and ZBG to find the correct position.^33^ The accommodation of the ZBG into the HDAC catalytic site is a crucial step of the inhibition process and is finely controlled by its zinc coordination ability and by key interactions with the surrounding protein residues. Moreover, the distance between the ZBG and the cap must be between 6.6 and 7.2 Å in order to have molecules with activity as HDAC inhibitors.^34^,^35^ These molecular features do not fit with amino acid conjugate coming from a partial metabolism of ADC **9**.

The putative anti-proliferative activity of conjugates was evaluated on two lung adenocarcinoma cell lines (NCI-H1975 and Calu-3), treated up to 6 days with ADC **8** or ADC **9**, as well as with equivalent doses of the parental antibody (free Ctx). As determined by cell proliferation curves, although at different extent ADC **8** inhibited tumour cell proliferation of both cell lines, showing IC_50_ value of 250 nM on NCI-H1975 cells and 40% of inhibition at the highest measured dose (500 nM) on Calu-3 cells. Although not extremely potent, conjugates **8** and **9** resulted much more active than Ctx alone that, at the same concentration, was not effective (Fig. S9†).

**Fig. 3:**
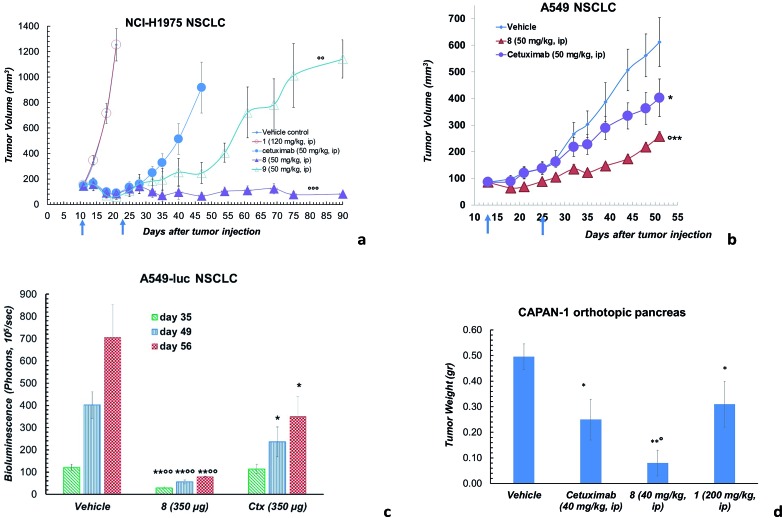
(a) Effects of ADC **8** and **9** in tumours developed in Nu/Nu mice after s.c. injection of 5 × 10^6^ NCI-H1975 cells. Lesion development and response to antibody treatment was monitored using a Vernier calliper. Mice injected i.p. with either **8**, **9**, Ctx (4 doses of 50 mg kg^–1^ once every 4 days) or **1** (4 doses of 120 mg kg^–1^ once every 4 days) and PBS (*n* = 8 mice/group; mean and SEM, °°°*p* < 0.001 and °°*p* < 0.01 *vs.* Ctx alone, Mann–Whitney's test). (b) Effects of ADC **8** in tumours developed in Nu/Nu mice for 13 days after s.c. injection of 5 × 10^6^ A549 cells. Lesion development and response to antibody treatment was monitored using a Vernier calliper. Mice injected i.p. with either **8** and Ctx (4 doses of 50 mg kg^–1^ once every 4 days) or PBS (*n* = 10 mice/group; mean and SEM, °*p* < 0.05 *vs.* Ctx; **p < 0.01 and *p < 0.05 *vs.* vehicle, Mann–Whitney's test. (c) Artificial metastatic lung cancer experiment carried out with 5 × 10^6^ A549-luc-C8 (A549luc) cells into the tail vein of immunodeficient SCID/beige mice. Tumour bioluminescence imaging (BLI) was recorded by Xenogen IVIS Imaging System 200, at different time points (+35, +49 and +56 days from cell injection), after i.p. injection of luciferin (150 μg per mouse). Mice were treated by aerosol with PBS or ADC **8** or Ctx (3.5 mL of 100 μg mL^–1^ solution) q7dx4 (*n* = 12 mice/group; mean and SEM, °°*p* < 0.01 *vs.* cetuximab; *p < 0.05 and **p < 0.01 *vs.* vehicle). (d) Orthotopic tumour pancreas experiment performed with 1 × 10^6^ tumor Capan-1 cells injected into pancreas. Tumor weight was evaluated 90 days after tumor injection. Mice treated intraperitoneally with **8** or Ctx (4 doses of 40 mg kg^–1^ once every 4 days), PBS and **1** (200 mg kg^–1^, q4dx4) (*n* = 10 mice/group); mean and SEM, °*p* < 0.05 *vs.* Ctx,*p < 0.05 and **p < 0.01 *vs.* vehicle).

However, ADC **8** and **9** showed a high antitumor activity in animal models (Fig. 3). Their efficacy in comparison with Ctx and **1** was evaluated in a mouse tumour xenograft model (non-small cell lung). The NCI-H1975 tumour cells were injected subcutaneously into nude Nu/Nu mice (day zero). By day 11, treatment was initiated when tumour lesions reached ∼100 mm^3^. Mice were randomized and injected with vehicle (PBS), **8**, **9**, **1** and Ctx (Fig. 3a). Mice treated with **8** showed absence of tumour in 50% of mice up to 90 days after the tumour injection. Although at a lower extent, also ADC **9** exhibited an anti-tumour activity, significantly higher than Ctx. No activity was observed with PBS or **1**, although the latter was used at high dose.^18^ Based on this result, ADC **8** was investigated in additional tumour models. In A549 NSCLC injected in nude Nu/Nu mice, the compound showed significant antitumor activity compared with Ctx (Fig. 3b). We were also delighted to observe that ADC **8** proved to be effective in a severe metastatic lung cancer model done by injecting type A549-luc-C8 (A549luc) cells into the tail vein of immunodeficient SCID/beige mice. After 1 week from tumour injection, animals were treated with ADC **8** or unconjugated Ctx by whole body aerosol (Fig. 3c). The evaluation of bioluminescence, analysed at three different times (35-49-56 days), showed that ADC **8** significantly inhibits tumour metastases with a higher potency in comparison with Ctx alone (Fig. 3c). Comparison among ADC **8**, **1** and Ctx was finally evaluated in a mouse model of the highly aggressive CAPAN-1 orthotopic pancreatic tumour. Treatments with ADC **8**, **1** and Ctx started 6 days after tumour inoculation and, 90 days after tumour injection, mice were sacrificed to analyse the pancreas tumour weight. ADC **8** showed to inhibit the tumour growth of 84% with 6 complete responses (6 pancreas free from tumour), while Ctx gave 50% of tumour growth inhibition with 2 complete responses.

**Fig. 4:**
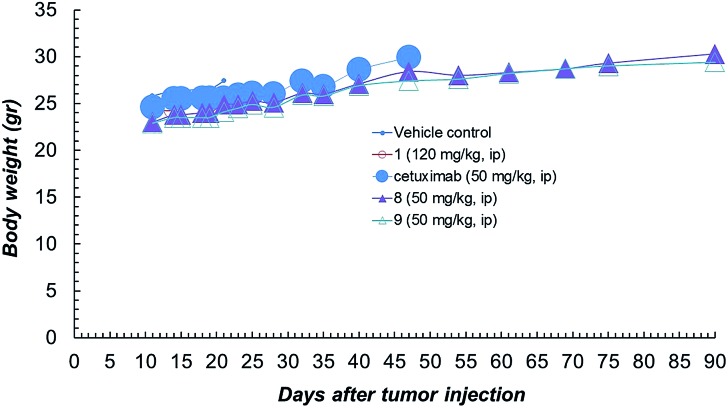
Body weight of NCI-H1975 tumor bearing mice, throughout the experiment described in Fig. 2a.

Compound **1** alone showed a lower activity on tumour growth (38%) although with five complete responses (Fig. 3d). Finally, it is worth noting that compound **8** showed the same toxicity than Ctx alone (Fig. 4). The body weight of mice treated with **8** was not affected throughout all the study duration, indicating that the treatment is well tolerated (Fig. 4).

*In vivo* combination study with Ctx and ST7612AA1 alone, demonstrated also that an equimolar mixture of the uncoupled drug and the antibody was well tolerated but not effective in the animal model. Overall, *in vitro* and *in vivo* results show that ADC **8** is a promising lead for further therapeutic applications and that its high activity is clearly due to conjugation of the HDAC inhibitor to the antibody.

## Conclusions

In conclusion, we have developed a new class of ADCs charged with HDAC inhibitors. Conjugates **8** (ST8154AA1) and **9** (ST8155AA1), the first example of an ADC for epigenetic modulation, delivered the HDAC inhibition to cells expressing the antibody antigens, inducing significant increment of histones 3 and 4 and α-tubulin acetylation. Animal models of human solid tumours indicate anti-tumour efficacy of such conjugates without the toxicity generally observed with traditional ADCs charged highly potent cytotoxic drugs. Overall, comparison of data suggests that ADC **8** is superior to **9**, probably due to the influence of the linker design and the release processes. With the preparation of ADCs **8** and **9** we have disclosed that it is possible to obtain active ADCs even with not highly cytotoxic warheads, with exceptional potential of decreasing the side effects of this class of drugs. This work clearly demonstrates that the paradigm ADC-cytotoxic payload can be overcome with many advantages for applications of ADCs beyond cancer therapy. Further work is in progress to understand why HDAC inhibitor payloads are different from numerous other tested in ADC field and to ascertain if the possibility to successfully conjugate medium/low cytotoxic drugs is limited to HDAC inhibitors or may be extended to other biologically active compounds.

## Live subject statement

In animal models, all the procedures adopted for housing and handling of animals were in strict compliance with Italian and European guidelines for Laboratory Animal Welfare. Studies were performed in accordance with the “Directive 2010/63/UE” on the protection of animals used for scientific purposes, made effective in Italy by the Legislative Decree 4 March 2014, no. 26, and ARRIVE guidelines 2.

## Conflicts of interest

G. G, L. V., F. M. M., A. M. A., G. B., R. D. S. are employees of Alfasigma srl. No conflict of interest is declared by all the other authors.

## Supplementary Material

Supplementary information

Supplementary information
