# Supplementary material for: Antibody drug conjugates (ADCs) charged with HDAC inhibitor for targeted epigenetic modulation
Source: Chem Sci. 2018 Jul 3;9(31):6490–6. doi: 10.1039/c7sc05266a (PMC6144071; doi:10.1039/c7sc05266a)
Supplement: Supplementary file 1 [file SC-009-C7SC05266A-s001.pdf]

Electronic Supplementary Material (ESI) for Chemical Science.
This journal is © The Royal Society of Chemistry 2018

Selected data obtained from hepatic microsome digestion of ADC **9**

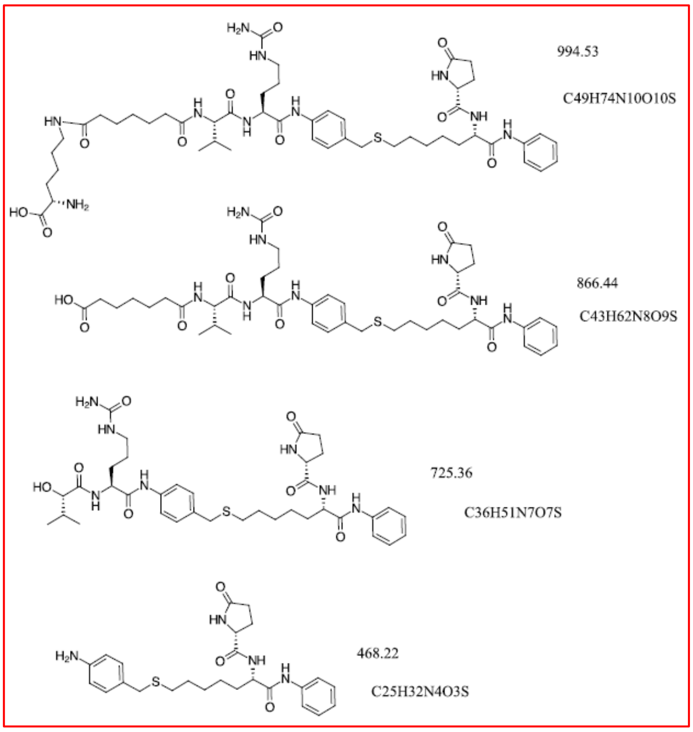

Possible expected metabolites.

Search for mass 467 $(M-H)^-$ Trace of Single ion current at T= 0, 24, 48 and 72 h respectively.

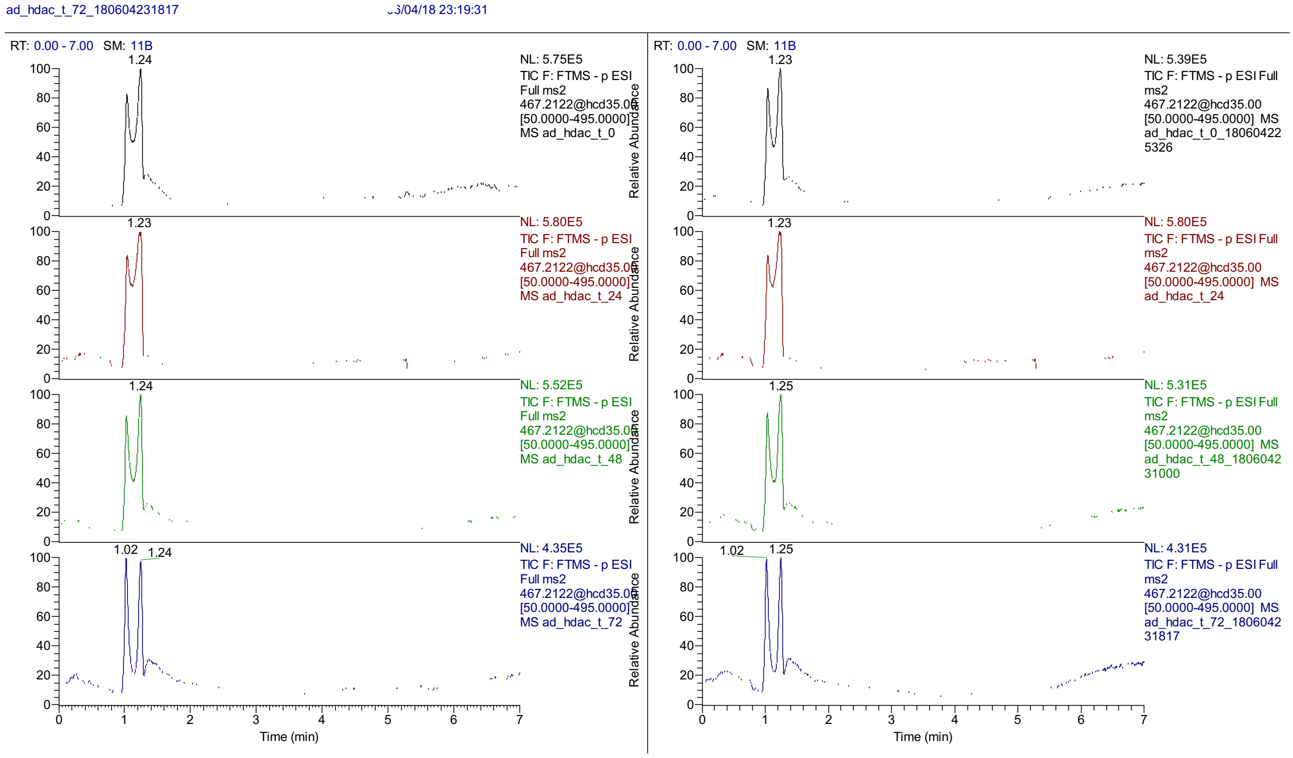

Search for mass 724, 865 and 993 $(M-H)^{.-}$ Traces of Single ion current at T= 0, 24, 48 and 72 h respectively

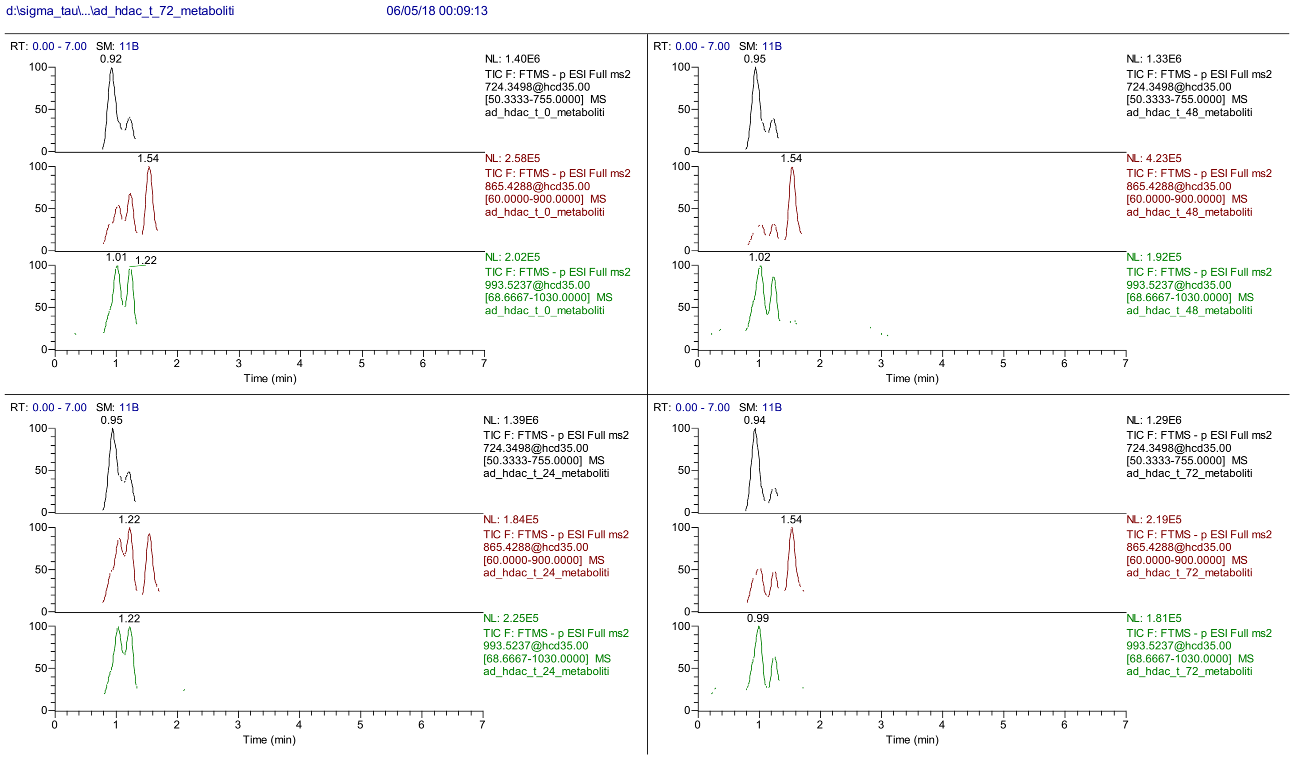

All the values of the peaks area are negligible or completely absent.
