# Supplementary material for: Antibody drug conjugates (ADCs) charged with HDAC inhibitor for targeted epigenetic modulation
Source: Chem Sci. 2018 Jul 3;9(31):6490–6. doi: 10.1039/c7sc05266a (PMC6144071; doi:10.1039/c7sc05266a)
Supplement: Supplementary file 2 [file SC-009-C7SC05266A-s002.pdf]

Electronic Supplementary Material (ESI) for Chemical Science.
This journal is © The Royal Society of Chemistry 2018

Antibody Drug Conjugates (ADC) Charged with HDAC Inhibitor for Targeted Epigenetic Modulation

Elena Cini, Valentina Faltoni, Elena Petricci, Maurizio Taddei,* Laura Salvini, Giuseppe Giannini,* Loredana Vesci, Ferdinando M. Milazzo, Anna Maria Anastasi, Gianfranco Battistuzzi, Rita De Santis

Supporting Information

General methods 2
Live subject statement 2
Synthesis of compound **3** 3
Synthesis of compound **4** 3
Synthesis of compound **6** 4
Synthesis of compound **7** 5
General procedure for the preparation of ADCs 5
General procedure for stability tests on compounds **4** and **7** 6
General procedure for stability tests of compounds **8** and **9** 8
ADC characterization 8
General biology procedures and in vivo experiments 9
Figure S1 (MALDI of conjugate **8**) 12
Figure S2 (MALDI of conjugate **9**) 12
Figure S3 (Size exclusion chromatography) 13
Figure S4 (FACS analysis) 14
Figure S5 (Immunoreactivity plot and affinity evaluation by BiaCore) 15
Figure S6 (Binding to SK-MEL-28 and Acetylation of HDAC-targeted proteins in SK-MEL-28) 16
Figure S7 (Acetylation of HDAC-target proteins in A549 cells) 17
Figure S8 (Acetylation of HDAC-target proteins in Capan-1 cells) 18
Figure S9 (Antiproliferative activity of compound **8** on NCI-H1975 and Calu-3 NSCLC) 19
Figure S10a (Calibration curve done at different concentrations of **2**). 20
Figure S10b (Time dependent formation of compound **2** from ADC **8** in the presence of human hepatic microsomes) 20
Figure S10c (Time dependent increment of the concentration of compound **2** after hepatic microsome digestion of ADC **8)** 21
Figure S11 (Comparison of MS spectra for **2** identity) 21
Figure S12 (Comparison of ^{1}H 600 MHz spectra of compound **4** in PBS) 22
Figure S13 (MetaSite analysis on a model derived from compound **7**) 23
Figure S14 (Tree of the most likely formed metabolites) 24
NMR spectra for product characterization 25

General methods

All reagents were used as purchased from commercial suppliers without further purification. The reactions were carried out in oven dried or flamed vessels. Solvents were dried and purified by conventional methods[1] prior use or, if available, purchased in anhydrous form. Flash column chromatography was performed with Merck silica gel 60, 0.040-0.063 mm (230-400 mesh). Merck aluminum backed plates pre-coated with silica gel 60 (UV254) were used for analytical thin layer chromatography and were visualized by staining with a $KMnO_4$ solution. NMR spectra were recorded at 25 °C and 400 or 600 MHz for ^{1}H and 100 or 150 MHz for ^{13}C. The solvent is specified for each spectrum. Splitting patterns are designated as s, singlet; d, doublet; t, triplet; q, quartet; m, multiplet; br, broad. Chemical shifts (δ) are given in ppm relative to the resonance of their respective residual solvent peaks. High and low resolution mass spectroscopy analyses were recorded by electrospray ionization with a mass spectrometer Q-exactive Plus. Melting points were determined in open capillary tubes and are uncorrected. HPLC/MS analysis were performed with the chromatographic LC/MSD system Agilent 1100 series, connected with UV detector (254 nm) using an Intersil ODS-3V C18 column (5 μm, 4.6 x 250mm), flow 0.8 mL/min, MeCN (0.1% HCOOH)/H_2O (0.1% HCOOH) gradient from 1:9 to 9:1 in 10 minutes. ESI ionization, flow of the drying gas (N2) 9L/min, temperature 350 °C, atomizing pressure 40 PSI, fragmentation 70 eV.

Live subject statement

In animal models, all the procedures adopted for housing and handling of animals were in strict compliance with Italian and European guidelines for Laboratory Animal Welfare. Studies were performed in accordance with the “Directive 2010/63/UE” on the protection of animals used for scientific purposes, made effective in Italy by the Legislative Decree 4 March 2014, n. 26, and ARRIVE guidelines.[2]

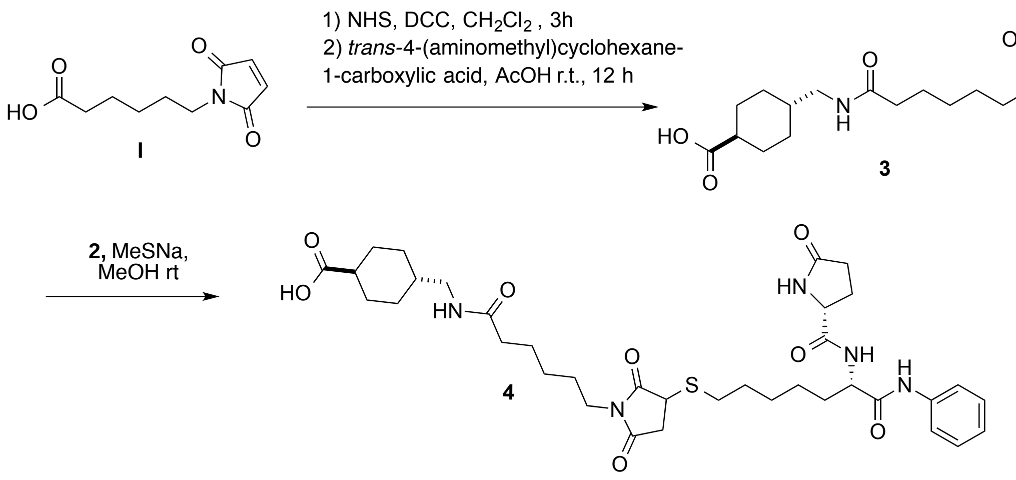

***trans*-4-{[6-(2,5-Dioxo-2,5-dihydro-1H-pyrrol-1-yl)hexanamido]methyl}cyclohexane-1-carboxylic acid (3).**
Dicyclohexylcarbodiimide (DCC) (2.68 g, 13.02 mmol) and N-hydroxysuccinimide (1.36 g, 11.84 mmol) are added at 0 °C to a stirrer solution of 6-maleimidohexanoic acid (**I**) (2.5 g, 11.84 mmol)[3] in anhydrous dichloromethane (15 mL) and the mixture stirred at room temperature for 3 hours. The white solid formed is filtered off with dichloromethane to remove the dicyclohexylurea, the organic phase is washed with HCl 0.1N and water, then dried over anhydrous sodium sulfate and the solvent removed by rotatory evaporation. The resulting residue is subjected to flash column chromatography with a medium pressure system Sepacore® Buchi (silica gel; gradient A: petroleum ether/B: ethyl acetate; B% 0-80 in 15 minutes) to give the activated acid as a white solid, m.p. 76-78 °C, lit[4] mp 79-80 °C, 2.46 g (67% yield). MS(ESI): m/z 309 $[M+H]^+$ The solid is dissolved into glacial acetic acid (20 mL) and *trans*-4-(aminomethyl)-cyclohexane carboxylic acid (2.38 g, 15.17 mmol) added at rt. The reaction mixture is stirred at room temperature for 16 hours. The acetic acid is removed under reduced pressure, the residue diluted with MeOH (15 mL) and filtered through a short pad of Celite. Water (50 mL) is slowly added to the MeOH solution and the mixture stirred at rt for 30 min. The solid is collected by filtration and dried under vacuum to give pure **3**, mp 152-154 °C. 1.48 gr (54% yield). MS (ESI): m/z 349 $[M-H]^-$ ^1H NMR (400 MHz, Methanol-d4) δ 6.85, (s, 2H), 3.54 (t, J = 7.2 Hz, 2H), 3.07 (d, J = 7.0 Hz, 2H), 2.27 – 2.21 (m, 2H), 2.05 (dd, J = 14.0, 3.5 Hz, 2H), 1.87 (dd, J = 13.3, 3.6 Hz, 2H), 1.69-1.62 (m, 4H), 1.49 – 1.32 (m, 6H), 1.05 (m, 2H). ^{13}C NMR (100 MHz, Methanol-d4) δ 178.04, 174.19, 170.74, 133.52, 44.59, 42.61, 36.80, 36.61, 35.03, 29.14, 28.05, 27.46, 25.55, 24.76. Anal calcd for $C_{18}H_{26}N_2O_5$: C, 61.70; H, 7.48; N, 7.99; O, 22.83; found C, 61.73; H, 7.49; N, 8.01.

4-{[6-(2,5-Dioxo-3-{[(6S)-6-{[(2R)-5-oxopyrrolidin-2-yl]formamido}-6-phenylcarbamoyl)hexyl]sulfanyl}pyrrolidin-1-yl)hexanamido]methyl}cyclohexane-1-carboxylic acid (4)
A solution of sodium thiomethylate 1 M in degassed methanol (17 mg, 0.25 mmol, 0.250 mL) is added at rt to a solution of **1** ST7612AA1 (100 mg, 0.25 mmol) in degassed methanol. The stirring mixture is maintained at rt for 30 min. This mixture is flushed with N_2 for 5-10 min to remove the formed MeSH and compound **3**

(88 mg, 0.25 mmol) is added. The mixture is kept at rt for 16 hours. The solvent is then removed by rotatory evaporation and the crude purified by flash column chromatography with a gradient 2-20% methanol in dichloromethane. Compound **4** is obtained as a white solid, mp: 88-90 °C, 102 mg (57%). MS(ESI): m/z 736 $[M+Na]^+$ ^{1}H NMR (400 MHz, Methanol-d4) δ 7.92 (s, 2H), 7.60 (d, J = 8.1 Hz, 2H), 7.34 (t, J = 7.8 Hz, 2H), 7.14 (t, J = 7.7 Hz, 1H), 4.55 (m, 1H), 4.34 (m, 1H), 3.86 (d, J = 9.0 Hz, 1H), 3.51 (t, J = 7.4 Hz, 2H), 3.21 (dd, J = 18.5, 9.1 Hz, 1H), 3.06 (d, J = 6.5 Hz, 2H), 2.82 (m, 2H), 2.59 – 2.09 (m, 8H), 2.08 – 1.77 (m, 6H), 1.76 – 1.23 (m, 16H), 1.02 (q, J = 12.9 Hz, 2H). ^{13}C NMR (100 MHz, Methanol-d4) δ 179.66, 178.38, 176.99, 175.26, 174.10, 173.05, 170.72, 137.62, 128.05, 123.66, 119.58, 77.65, 56.20, 53.54, 44.62, 42.79, 38.69, 37.78, 36.79, 35.30, 31.41, 30.37, 29.18, 28.78, 28.15, 28.08, 27.47, 26.47, 25.53, 25.03, 24.75, 24.60. Anal calcd for $C_{36}H_{51}N_5O_8S$: C, 60.57; H, 7.20; N, 9.81; O, 17.93; S, 4.49; found C, 60.61; H, 7.22; N, 9.83.

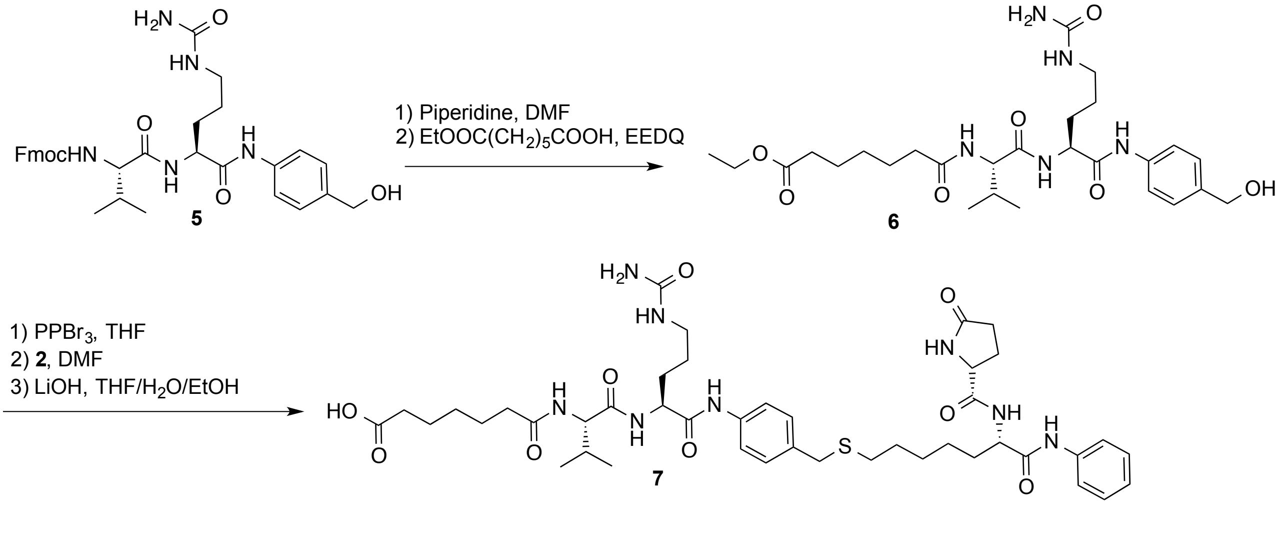

Ethyl 7-(((S)-1-(((S)-1-((4-(hydroxymethyl)phenyl)amino)-1-oxo-5-ureidopentan-2-yl)amino)-3-methyl-1-oxobutan-2-yl)amino)-7-oxoheptanoate (6)

Piperidine (215 μL, 2.18 mmol) is added to a solution of compound **5**[5] (660 mg, 1.09 mmol) in anhydrous DMF (5 mL) and the reaction stirred at rt for 3 h. The solvent is removed under vacuum and the residue treated with dichloromethane to obtain a solid (218 mg) that is dissolved in a mixture of dichloromethane/methanol 2:1 (20 mL). Monoethyl pimelate (0.123 mL, 0.69 mmol) is added followed by EEDQ (284 mg, 1.14 mmol) and the reaction stirred in the dark at rt for 16 hours. The solvents are removed under vacuum and the residue purified by flash column chromatography with a gradient 2-20% methanol in dichloromethane to give product **8** as a waxy solid, 170 mg (54% yield). ^{1}H NMR (400 MHz, Methanol-d4) δ 7.59 (d, J = 8.1 Hz, 2H), 7.34 (d, J = 8.3 Hz, 2H), 4.60 – 4.56 (m, 3H), 4.25 (d, J = 7.2 Hz, 1H), 4.13 (m, 2H), 3.36 – 3.13 (m, 2H), 2.34 (m, 3H), 2.12 (m, 1H), 1.90 (m, 1H), 1.83-1.75 (m, 1H), 1.65 (m, 7H), 1.35 (m, 2H), 1.28 (m, 3H), 1.01 (m, 6H). ^{13}C NMR (151 MHz, DMSO-d6) δ 173.32, 172.82, 171.73, 170.85, 159.35, 138.00, 137.84, 127.36, 119.26, 63.04, 60.11, 58.10, 53.50, 35.44, 33.88, 29.82, 28.54, 27.26, 25.53, 24.69, 19.72, 18.67, 14.60. HRMS (ESI) calcd for $C_{27}H_{43}NaN_5O_7$ $[M+Na]^+$ 572.3061; found 572.3058.

6-{[(1S)-1-{[(1S)-4-(Carbamoylamino)-1-{[4-({[(6S)-6-{[(2R)-5-oxopyrrolidin-2-yl]formamido}-6-(phenylcarbamoyl)hexyl]sulfanyl}methyl)phenyl]carbamoyl}butyl]carbamoyl}-2-methylpropyl]carbamoyl}hexanoic acid (7)

Phosphorus tribromide (0.043 mL, 0.46 mmol) is added at 0° C to a solution of compound **6** (170 mg, 0.31 mmol) in anhydrous THF (5 mL). The mixture is kept at 0° C for 3 hours. The reaction mixture is rapidly filtered through a short path of silica gel and the solvent evaporated. The crude (MS(ESI): m/z 612, 614 $[M+H]^+$; 634, 636 $[M+Na]^+$) is dissolved in the minimum amount of anhydrous DMF and product **2** (prepared as described for the synthesis of compound **4, (**112 mg, 0.31 mmol) added in degassed methanol (8 mL) and further evaporated under reduced pressure until complete MeOH removal. The reaction is stirred at room temperature for 16 hours. The solvent is removed by rotatory evaporation and the crude purified by flash column chromatography with a gradient 1-40% methanol in dichloromethane to give the ethyl ester of **7** as an amorphous solid; yield, 146 mg (55% yield). MS(ESI): m/z 896 $[M+H]^+$; 918 $[M+Na]^+$ ^1H NMR (400 MHz, Methanol-d4) δ 7.60 (s, 4H), 7.43 – 7.18 (m, 4H), 7.13 (s, 1H), 4.55 (m, 4H), 4.40 (m, 2H), 4.13 (m, 2H), 3.66 (m, 6H), 3.19 (s, 4H), 2.75 (s, 2H), 2.37 (m, 6H), 2.11 (s, 2H), 1.97 (s, 1H), 1.82 (s, 2H), 1.64 (s, 7H), 1.42 (s, 4H), 1.27 (s, 4H), 1.01 (s, 6H). The product (146 mg, 0.16 mmol) is dissolved in a mixture of THF/water/ethanol 1:1:1 (6 mL). Lithium hydroxide monohydrate (42 mg, 1 mmol) is added and the reaction stirred at room temperature for 6 hours. The mixture is treated with HCl 1N (5 mL) and further extracted with ethyl acetate (20 mL) and washed with brine (5 mL). The organic phase is separated and, after drying over anhydrous Na_2SO_4 the solvent evaporated to give crude **7** as a solid pure for the use in the next step, m.p. 211-212 °C. 136 mg, 95%. An analytical sample was purified by flash column chromatography with methanol : dichloromethane 1 : 10. ^1H NMR (400 MHz, DMSO-d6) δ 10.10 (s, 1H), 9.95 (s, 1H), 8.22 (d, J = 7.9 Hz, 1H), 8.07 (d, J = 7.9 Hz, 1H), 7.86 (m, 2H), 7.62 (d, J = 8.0 Hz, 2H), 7.56 (d, J = 7.0 Hz, 2H), 7.34 (m, 2H), 7.24 (d, J = 9.0 Hz, 2H), 7.08 (t, J = 7.1 Hz, 1H), 6.00 (bs, 1H), 5.42 (s, 2H), 4.45 (m, 2H), 4.21 (m, 2H), 3.67 (m, 2H), 2.98 (m, 2H), 2.65 (m, 2H), 2.40 2.18 (m, 6H), 2.14 – 2.11 (m, 2H), 1.65 – 1.34 (m, 20 H), 0.87 (m, 6H). ^{13}C NMR (150 MHz, DMSO-d6) δ 177.87, 172.87, 171.73, 171.10, 170.98, 159.48, 139.36, 138.08, 133.85, 129.51, 129.14, 123.80, 119.74, 119.51, 58.22, 55.94, 55.79, 53.96, 53.81, 53.54, 35.53, 35.08, 32.50, 30.92, 30.89, 29.75, 29.70, 29.66, 29.01, 28.73, 28.32, 27.15, 26.06, 25.64, 25.62, 25.39, 24.90, 19.73, 18.72. HRMS(ESI) calcd for $C_{43}H_{62}N_8O_9SNa$ $[M+Na]^+$ 889.4259 (100%), 890.4292 (46%); found 889.4261 (100%), 890.4293 (44%).

General procedure for the preparation of ADCs through conjugation via ε-amino groups of lysine residues. Conjugate 9 (Ctx-NH-7)

DCC (7 mg, 0.035 mmol) and N-hydroxysuccinimide (3.4 mg, 0.03 mmol) are added at room temperature to a stirrer solution of compound **7** (18 mg, 0.020 mmol) in anhydrous DMF (0.5 mL). The mixture is stirred at room temperature for 16 hours. The white solid formed in this reaction is filtrated and the solvent removed

under vacuum. The resulting residue (MS(ESI): m/z 987 $[M+Na]^+$) is dissolved in DMSO. Contemporary, a Ctx solution is buffer exchanged using a 10 kDa cutoff dialysis membrane to yield antibodies solution in PBS pH 7.4 and to remove interfering preservative (glycine). The Ctx concentration after dialysis is determined measuring the OD_{280} and the observed absorbance is divided by 1.35. A 10 mM solution of **7** activated as NHS ester is prepared in DMSO and a 20-fold molar excess of this solution added to the dialyzed antibody solution. The reaction is incubated at room temperature, with gentle continuous mixing and after 1 hour, quenched with a 20 mM glycine aqueous solution. The final product is dialyzed in PBS overnight at 4°C using a 10 kDa cutoff membrane in order to remove the excess of unreacted payload. The DAR is determined by MALDI mass spectrometry, using an Ultraflex III mass spectrometer (Bruker, GmbH), operating in positive linear mode. Briefly, 100 µl of unconjugated antibodies and product **9** are desalted using PD spin trap G25 (GE Healthcare) eluting in water. A 10 mg/ml s-DHB MALDI matrix solution is prepared in 0.1%TFA dissolved in H_2O : acetonitrile (50:50, v/v). A sample solution of Ctx or **9** (2 µL) are deposited on MALDI target using a double layer sample deposition method. The mass spectra are acquired in a mass to charge range starting from 50 kDa to 180 kDa. The mass difference between unconjugated and conjugated antibodies used to determine the DAR, in more than 10 experiment, is 5 ± 2.

General procedure for stability tests on compounds 4 and 7.

PBS buffer 20 mM was used for incubation at pH 7.4, HCl buffer for incubation pH 2.6 and Gly/NaOH buffer for incubation pH 10.4 and the products analyzed using the same HPLC/MS method.

A 100 mM DMSO solution of **4** was diluted with the proper buffer solution containing 10 mM GSH to obtain a 3 mM solution. The mixture was then incubated at 37 °C and aliquots were periodically removed and analyzed by HPLC and HPLC/MS (5 µm, 4.6 x 250mm, 20 cm C-18 column, Intersil ODS-3V, MeCN (0.1% HCOOH)/H_2O (0.1% HCOOH) from 10:90 to 90:10 in 10 min, then 90:10 for 20 min at 0.8 mL/min, 254 nm).

Compound **4** in PBS or mouse plasma

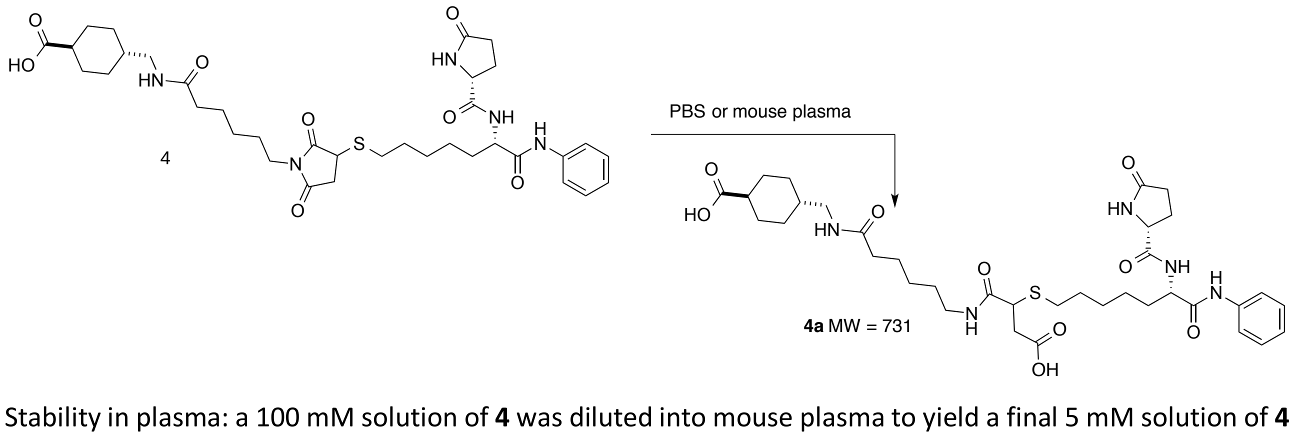

Stability in plasma: a 100 mM solution of **4** was diluted into mouse plasma to yield a final 5 mM solution of **4** in plasma. Samples were incubated at 37 °C under 5% CO_2, and aliquots collected at 3 time points (0, 5 h, 24

h and 72 h) and injected in HPLC/MS following the previous described procedure. Single ion current analysis showed the presence of **4a** [m/z = 730, $(M\text{-}H)^-$] and no trace of **2** [m/z = 362, $(M\text{-}H)^-$]

Stability in PBS**.** Single ion current analysis showed the presence of **4a** [m/z = 730, $(M\text{-}H)^-$] and no trace of **2** [m/z = 362, $(M\text{-}H)^-$]. 600 MHz ^1H NMR was also recorded after 1.5; 6 and 12 h that showed a new signal at 4.23 ppm (dd) ascribable to the CH carrying the S of the new formed succinimide acid **4a**. This signal increased after 12 h of incubation.

General procedure for stability tests of compounds 8 and 9 in the presence of human hepatic microsomes.[6]

Mixed-gender human liver S9 fraction (Sigma-Aldrich, 20 μL) was mixed with the proper buffer (130 μL of AcONa for pH 5.5 or PBS for pH 7.4) and, after incubation at 37 °C, a portion of ADC **8** or **9** (50 μL of a 20 μM DMSO solution) was added. After a carful mixing, a sample (50 μL) was collected at t = 0, 24 48 and 72 h respectively. The sample was mixed with cool MeCN (250 μL) with a vortex and centrifuged at 10000 rpm for 10 min. The supernatant was transferred in a vial and dried under a dry N_2 flux. The residue was finally reconstituted with 5% MeCN in H_2O. 10 μL of this solution was analyzed by HPLC-ESI-MS/MS. The MS/MS spectra were recorded at 17500 resolution in negative charged ESI (see page S21).

The quantitative data was based on a dilution curve of compound **2** (intensity of mass 235/concentration, Fig S10a). Formation of **2** from **8** was time dependent, after 72 h the concentration of 2 was 11.8 μM. As the starting concentration of ADC **8** was 5 μM, DAR = 8, corresponding to a 40 μM concentration of the drug, the release after 72 h was 29% (Fig S10b and S10c).

From **9**, no signal attributable to the catabolism of the ADC construct was detected. In the following scheme the mass of potential fragment investigated are reported

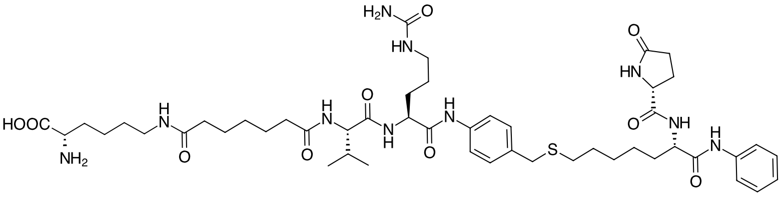

994.53

$C_{49}H_{74}N_{10}O_{10}S$

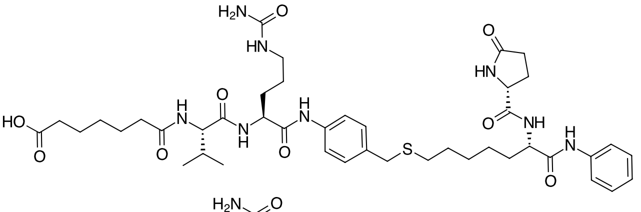

866.44

$C_{43}H_{62}N_8O_9S$

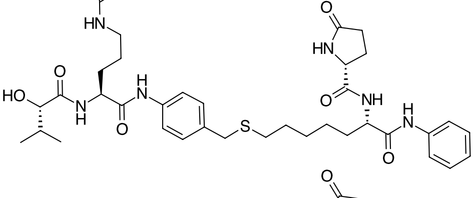

725.36

$C_{36}H_{51}N_7O_7S$

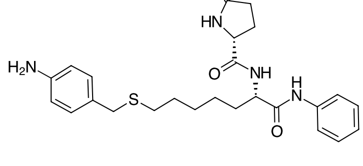

468.22

$C_{25}H_{32}N_4O_3S$

Stability in plasma: ADC **8** or **9** (50 μL of a 20 μM DMSO solution) was added into mouse plasma and the samples were incubated at 37 °C under 5% CO_2. Aliquots were after 6, 24 and 72 h and injected in HPLC/MS following the previous described procedure. The presence of the peak at m/z 328.1667 was never detected.

ADC characterization

The purity and integrity of ADCs **8** and **9** were confirmed by SEC-HPLC on TSKgel G3000 SWXL (7.8 x 300 mm) column (Tosoh Bioscience). Immunoreactivity was tested by antigen-specific ELISA. Briefly, Immuno MAXISORP 96-well plates (Nunc) were coated overnight at 4°C with 50 ng/well of recombinant human EGF-R/Erb1 Fc chimera (R&D). Plates were washed with PBS 0.1% Tween-20 (PBS-T), blocked with PBS-T 1% BSA for 2 h, and incubated with serial dilutions of antibodies, 1 h at room temperature. After washings, anti-human K light chain horseradish peroxidase (HRP)-conjugated antibody (Sigma Aldrich), diluted 1:1,000 in blocking solution, was added 1 h at room temperature. After washings, 200 µL/well TMB substrate (Sigma Aldrich) were added and plates incubated 30 min at 37°C. The reaction was blocked by adding 100 µL/well of 0.5M H_2SO_4 solution, and optical density at 450 nm was measured by ELISA reader (TECAN). The potential presence of endotoxins was tested by LAL test (Endosafe PTS/MCS Cartridges).

General biology procedures and in vivo experiments

Cell lines

NCI-H1975, Capan-1 and SKMel28 cell lines were from ATCC; A549 cells were from DSMZ. NCI-H1975 and A549 were cultivated in RPMI-1640 medium 10% FBS whereas Capan-1 cells were cultivated in RPMI-1640 medium 20% FBS. SKMel28 cells were cultivated in DMEM 10% FBS, supplemented with non- essential amino acids.

ADC tumor cell proliferation

The effect of ADCs on cell proliferation was evaluated on two lung adenocarcinoma cell lines (NCI-H1975 and Calu-3). More in details, NCI-H1975 tumor cells are characterized by overexpression of double-mutant (L858R, T790M) *ErbB1* gene, whereas Calu-3 express the wild type form of EGFR but mutant K-Ras (G13D) gene, as well as mutant TP53 and CDKN2A genes.

Cells were seeded (at 3.000-5.000 cells/well) into 96-well plates in complete culture medium and then incubated for 6 days, in quadruplicate, with scalar concentrations of ADCs, ranging from 500 to 6.25 nM. Inhibition of cell proliferation was measured by CellTiter-Glo Luminescent Cell Viability Assay (Promega), through a Veritas luminometer (Promega). Data were expressed as the average (± SE) of percentage inhibition of two independent experiments. The IC_{50} values were ultimately calculated by using the GraphPad Prism 5.02 software.

ADC binding (FACS Analysis)

Pellets of NCI-H1975, A549, Capan-1 or SKMel28 cells were incubated 1 h at 4°C with Ctx or ADCs (5 µg/mL in 100 µL). After washings, cells were incubated with mouse anti-human PE (or FITC)-conjugated Ig (BD). Cytofluorimetry was performed with FACScalibur (BD).

High content screening (HCS) fluorescence imaging

Cells were seeded in 96-well microtiter plates in complete medium and then incubated with Ctx or ADCs **8** and **9**, for the indicated times. After cell fixation with 4% formaldehyde in PBS, permeabilization with 0.2% Tween-20 in PBS (PBS-T) and blocking with 2% BSA in PBS-T, Ctx or ADCs **8** and **9** were detected by FITC (or PE)-conjugated mouse anti-human Ig (BD).

Expression of protein targets after cell fixation, permeabilization and blocking as described above, was evaluated by adding the following specific primary antibodies: rabbit anti-acetyl-Histone H3 (Lys9/Lys14) (Cell Signaling), rabbit anti- acetyl-Histone H4 (Ser1/Lys5/Lys8/Lys12) (Santa Cruz), or mouse anti acetyl-tubulin (6-11B-1) (Santa Cruz). FITC-conjugated goat anti-rabbit or goat anti-mouse IgG (BD) were then added, according to the primary antibody used. Cells were counterstained with Draq5 dye (Cell Signaling). Fluorescence signals were acquired by the High Content Screening (HCS) system Operetta (Perkin Elmer) and images analyzed through Harmony software (Perkin Elmer).

Western blotting

A549 cells were seeded in 10-cm culture plates in complete medium, and then cultivated with 20 µg/mL Ctx or ADCs, for 3 hours. Cells were then washed twice, and whole cell lysates were prepared by incubation, 10 min on ice, with 1× Lysis Buffer (Cell Signaling) supplemented with protease inhibitors. Cell lysates were subjected to sonication prior to centrifugation at 14.000 × g, for 10 min at 4°C, to remove cell debris. Protein content was determined by Bradford method. Equal amounts of soluble proteins were separated on SDS-PAGE and then transferred to nitrocellulose membrane (Amersham Hybond-ECL; GE Healthcare). Membranes were blocked 3 hours at room temperature with 5% non-fat dry milk in PBS 0.05% Tween-20 (PBS-T) before overnight incubation, at 4°C, with one of the following primary antibodies: rabbit anti- acetyl-Histone H4 (Ser1/Lys5/Lys8/Lys12) (Santa Cruz) or mouse anti acetyl-tubulin (6-11B-1) (Santa Cruz). Immunoblotting with mouse anti-β-actin antibody (Sigma Aldrich) was performed to normalize protein loading. After washings with PBS-T, membranes were incubated 1 hour with the appropriate secondary HRP-conjugated anti-rabbit or anti-mouse IgGs (Sigma Aldrich and Amersham GE-Healthcare, respectively). Immunoreactive bands were visualized by enhanced chemiluminescence detection and analyzed through phosphoimaging (STORM, Molecular Dynamics) or by exposure to X-ray film (Amersham Hyperfilm ECL; GE-Healthcare).

In vivo tumor models

In animal models, all the procedures adopted for housing and handling of animals were in strict compliance with Italian and European guidelines for Laboratory Animal Welfare. All the experiments were carried out using female athymic nude mice, 5-6 weeks-old (Harlan or Charles River Laboratories).

The ADCs **8** and **9** (ST8154AA1 and ST8155AA1 respectively) and Ctx were dissolved in PBS before to be delivered to mice, the HDACi **1** (ST7612AA1) in solutol HS15 (1:20 v/v in PBS). In NCI-H1975 tumor xenograft model, tumor cells ($3x10^5$/mouse) were injected subcutaneously into nude Nu/Nu mice (day zero) followed by caliper measurements twice a week. By day 11, treatment was initiated when tumor lesions reached ~100 mm^3. Mice were randomized and injected with vehicle (PBS), **8** (ST8154AA1), **9** (ST8155AA1) and Ctx (4 doses, 50 mg/kg, every 4 days, i.p.) or **1** (ST7612AA1) (4 doses, 120 mg/kg, ip, q4dx4). All the compounds revealed a good tolerability as shown for body weight (Figure S9).

In A549 NSCLC s.c. injected in nude nude Nu/Nu at $5x10^6$ tumor cells (10 mice/group), the compound **8** and Ctx were given at 50 mg/kg, ip, q4dx4, starting 13 days after tumor injection, measured by digital caliper. Tumor volume (TV) was calculated using the formula: TV (mm^3) = [d2 x D]/2, where d and D are the shortest and the longest diameter, respectively. The efficacy of the drug treatment was assessed as: TV inhibition percentage (TVI%) in treated versus control mice, calculated as: TVI%=100-(mean TV treated/mean TV

control x100). When tumors reached a volume of 500- 1000 mm^3, mice were sacrificed by cervical dislocation. To examine the possible toxicity of treatment, body weight was recorded throughout the study.

In the artificial metastatic lung cancer carried out with $5x10^6$ A549-luc-C8 (A549luc) cells into the tail vein of immunodeficient SCID/beige mice (12 mice/group), compound **8** or Ctx (3.5 mL of 100 µg/mL solution) were given after 1 week the tumor injection by whole body aerosol (by means of the AirFamily system, Pic indolor) according to the schedule q7dx4. Tumor bioluminescence imaging (BLI) was recorded at different time points by Xenogen IVIS Imaging System 200 (Perkin Elmer), 15 min after i.p. injection of luciferin (150 µg/mouse).

The ADC **8** was also evaluated in comparison with Ctx at 40 mg/kg, ip (q4dx4) and compound **1** at 200 mg/kg, ip, q4dx4 on CAPAN-1 orthotopic pancreas. $1x10^6$ tumor cells were suspended in 15 µL and injected in animals previously anesthetized with avertin 2.5%. Skin was cut for 1.5 cm, pancreas was exposed to inject tumor cells inside the pancreas for 10 seconds. Pancreas was placed back into the original position and the peritoneum was sutured. All the treatments were started 6 days after tumor inoculation. All the statistical analyses to compare different experimental groups were carried out by Mann-Whitney's test.

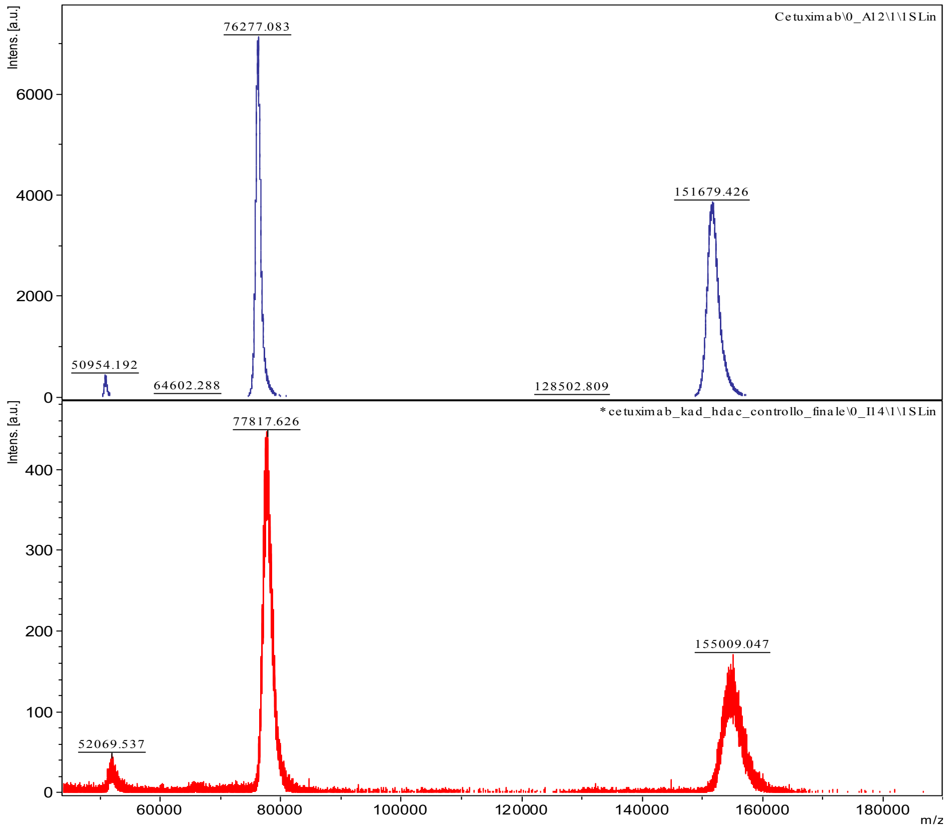

Figure S1: Comparison of MALDI spectra of Ctx alone (blue line, up;) and conjugate **8** (red line; down)

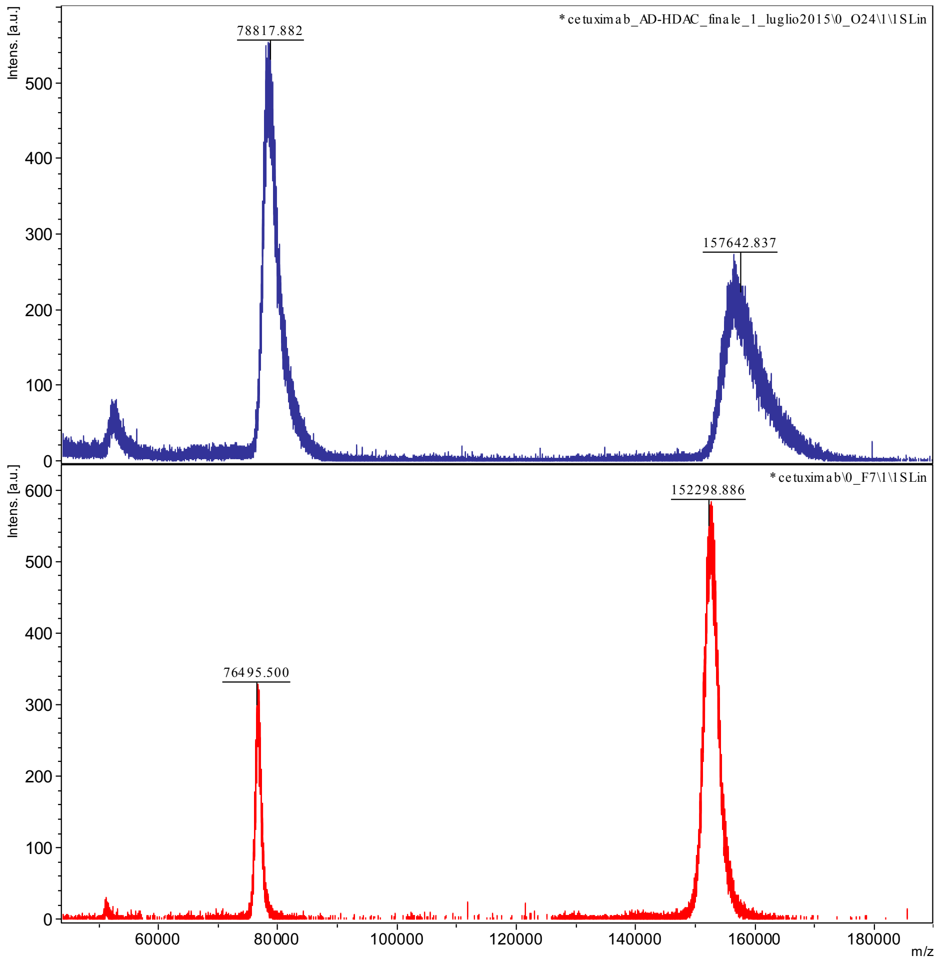

Figure S2: Comparison of MALDI spectra of Ctx alone (red line; down) and conjugate **9** (blue line; up)

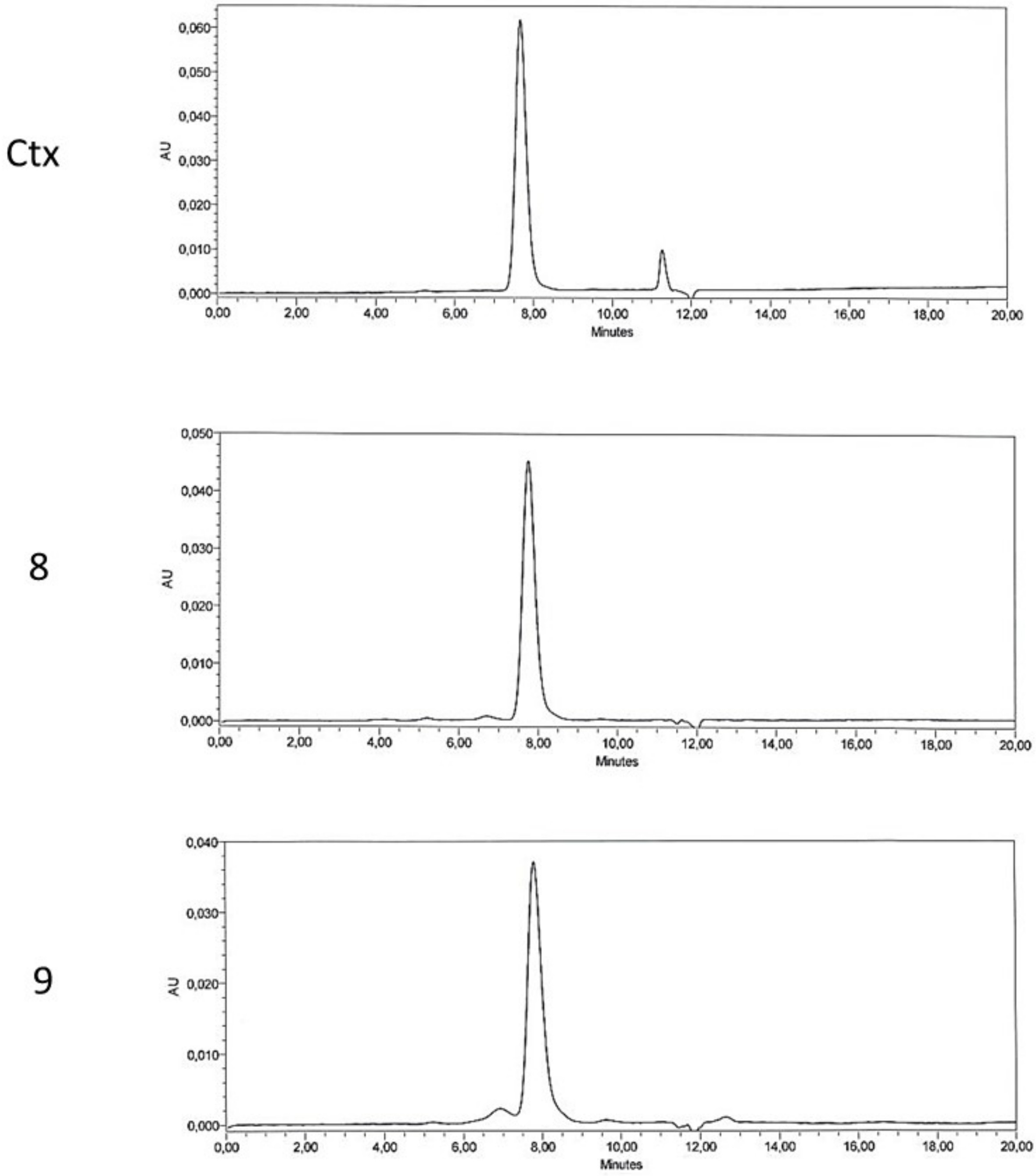

Figure S3: Size exclusion chromatography of Ctx and compounds **8** and **9**.

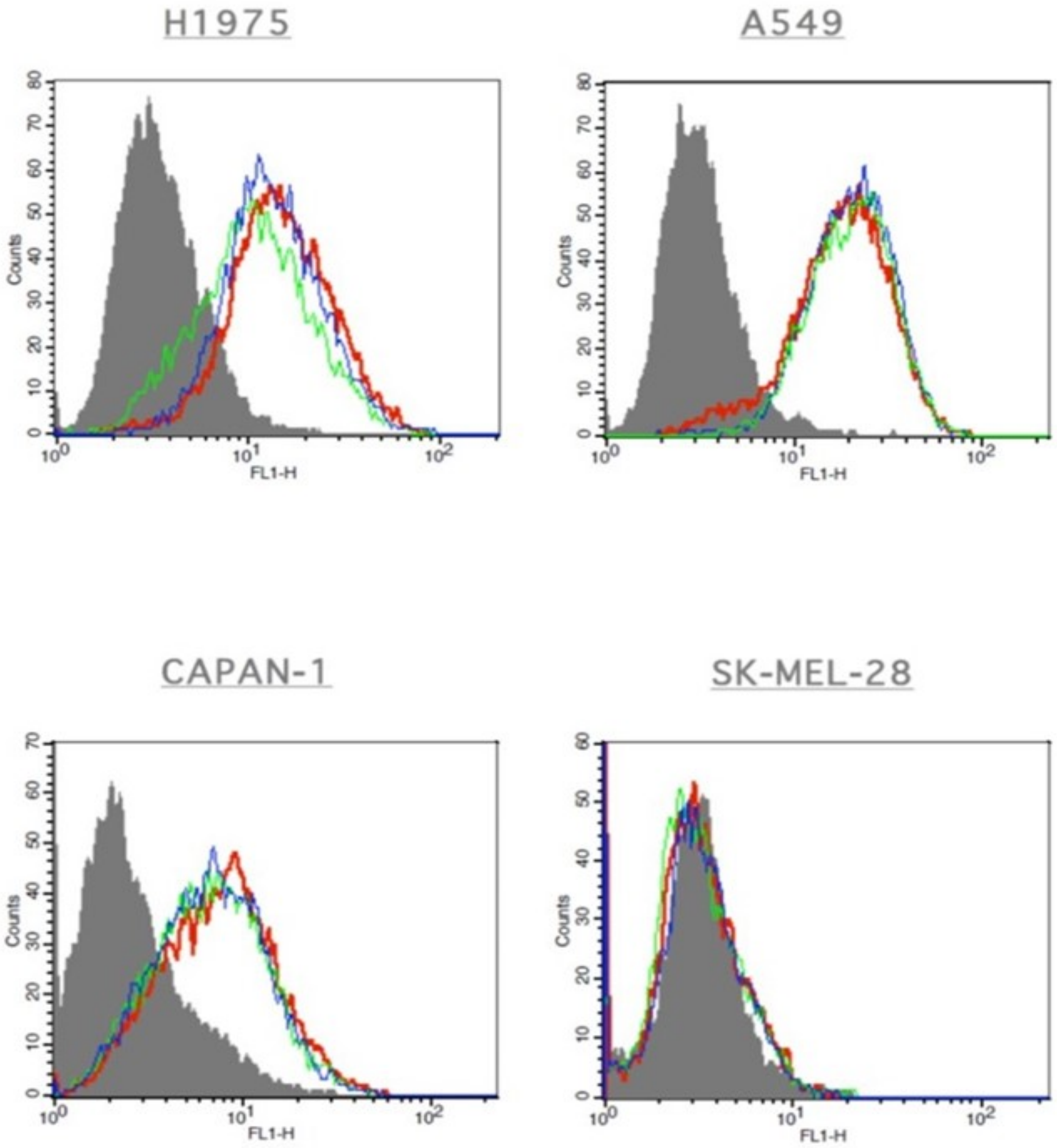

Figure S4: FACS analysis of native Ctx (red line) and conjugates **8** (green line) and **9** (light blue line) to Capan-1 (human pancreas carcinoma), H1975 and A549 (human lung carcinoma) or SK-MEL-28 (human melanoma) cell lines. Cell pellets were incubated with test compounds (10 µg/mL in PBS), and then stained with FITC-conjugated mouse anti-human Ig and propidium iodide. Grey peaks refer to cells without primary antibody.

a

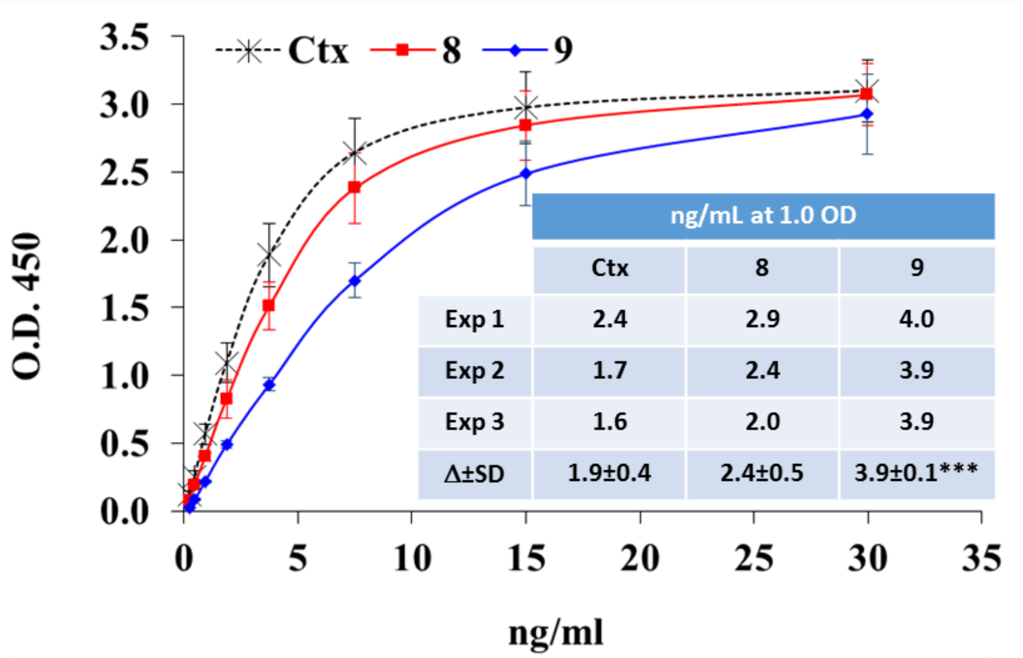

ng/mL at 1.0 OD			
	Ctx	8	9
Exp 1	2.4	2.9	4.0
Exp 2	1.7	2.4	3.9
Exp 3	1.6	2.0	3.9
Δ±SD	1.9±0.4	2.4±0.5	3.9±0.1***

b

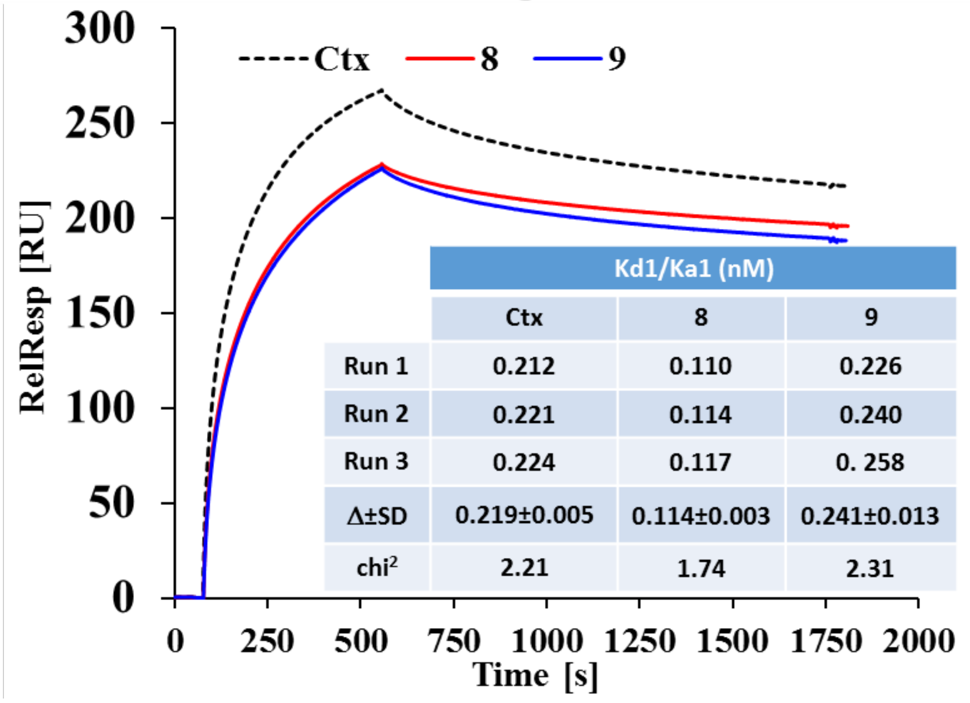

Kd1/Ka1 (nM)			
	Ctx	8	9
Run 1	0.212	0.110	0.226
Run 2	0.221	0.114	0.240
Run 3	0.224	0.117	0. 258
Δ±SD	0.219±0.005	0.114±0.003	0.241±0.013
chi^2	2.21	1.74	2.31

Figure S5: a, Immunoreactivity of conjugates **8** and **9** compared to Ctx, tested by antigen-specific ELISA. Binding to immobilized recombinant EGF-R/ErbB1 Fc chimera. Detection through anti-human k light chain horseradish peroxidase (HRP)-conjugated antibody and TMB substrate. Optical density at 450 nm measured by ELISA spectrophotometer. Results are the mean (±SD) of three independent experiments. Inset table shows ng/mL concentrations to get 1.0 OD. Statistical analysis by Student's T test ****p*< 0.001 **b,** Affinity evaluation by Surface Plasmon Resonance (BiaCore T200). Recombinant human EGF-R/ErbB1R His Tag immobilized on a CM5 sensor chip by amine coupling. Cetuximab and derivatives **8** and **9** concentrations ranging from 0.4 to 33.3 nM were flowed (33.3 nM curves showed in figure) and sensorgrams analyzed by BiaEvaluation 1.0 software. Best fitting obtained applying a bivalent analyte model. Inset table shows Kd1/Ka1 ratios. Results are the mean (±SD) of three sample runs.

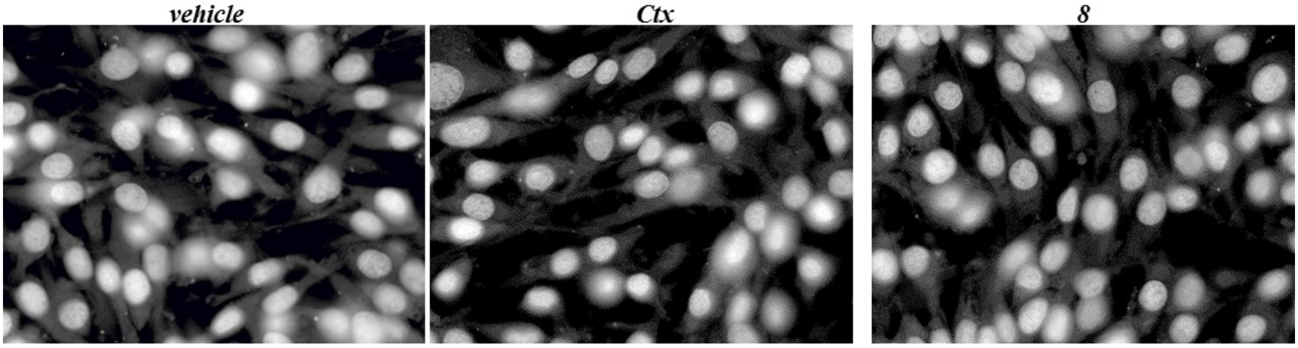

a

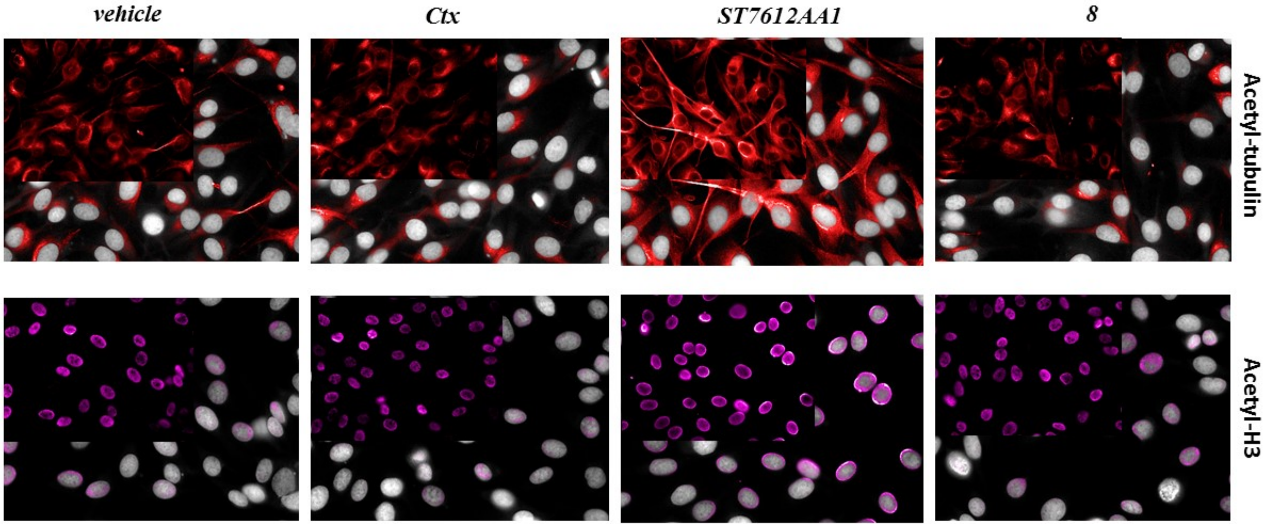

b

Figure S6: **a)** Binding of Ctx and ADC 8 to SK-MEL-28 ($EGFR^-$ melanoma human cells); **b)** Effect on acetylation of HDAC-targeted proteins in SK-MEL-28 cells. ST7612AA1 was used as positive control. Insets show specific fluorescence signals within the cells. Each image is representative of at least 5 fields of duplicate wells. Magnification 60x. Data are from one representative experiment out of two.

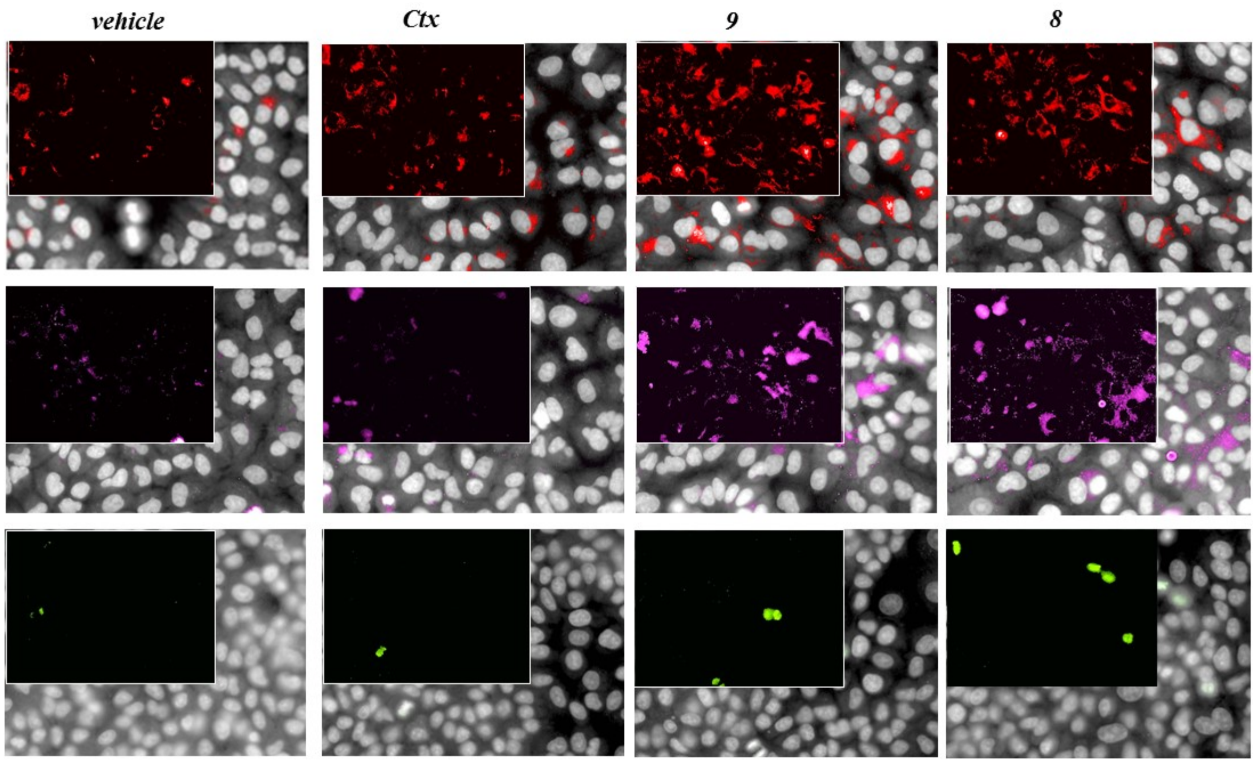

Figure S7: Effect of native Ctx, ADCs **8** (ST8154AA1) or **9** (ST8155AA1), on acetylation of HDAC-target proteins in A549 (human lung carcinoma) cells. Cells were cultivated 3 hours at 37°C with antibodies (5 µg/mL). After two washings, cells were fixed and stained with anti-acetylated-α-tubulin (red signal), anti-acetylated histone H3 (pink signal) or anti-acetylated histone H4 (green signal) IgG and then with FITC-conjugated secondary antibodies. Draq5 dye staining of nucleus and cytoplasm. Insets show fluorescence signals specifically associated to acetylated proteins. Fluorescence imaging by High Content Screening (HCS) Operetta. Each image is representative of at least 5 fields of duplicate wells. Magnification 60x. Data are from one representative experiment out of two.

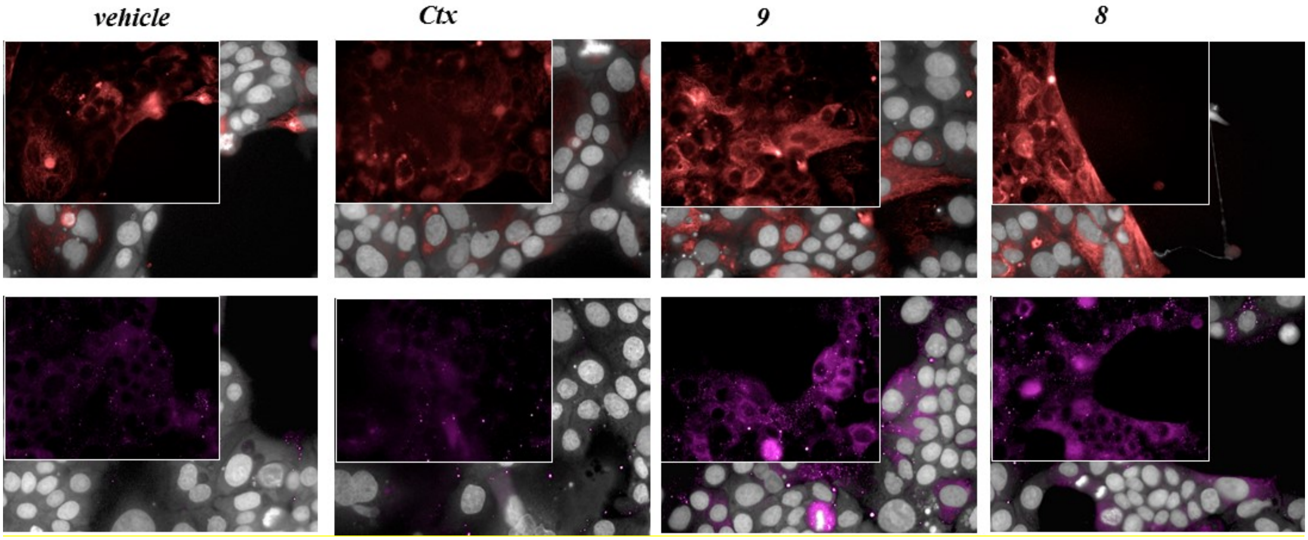

Figure S8: Effect of native Ctx, ADCs **8** (ST8154AA1) or **9** (ST8155AA1), on acetylation of HDAC-target proteins in Capan-1 (human pancreas carcinoma) cells. Cells were cultivated 3 hours at 37°C with antibodies (5 µg/mL). After two washings, cells were fixed and stained with anti-acetylated-α-tubulin (red signal) or anti-acetylated histone H3 (pink signal) IgG and then with FITC-conjugated secondary antibodies. Draq5 dye staining of nucleus and cytoplasm. Insets show fluorescence signals specifically associated to acetylated-proteins. Fluorescence imaging by High Con-tent Screening (HCS) Operetta. Each image is representative of at least 5 fields of duplicate wells. Magnification 60x. Data are from one representative experiment out of two.

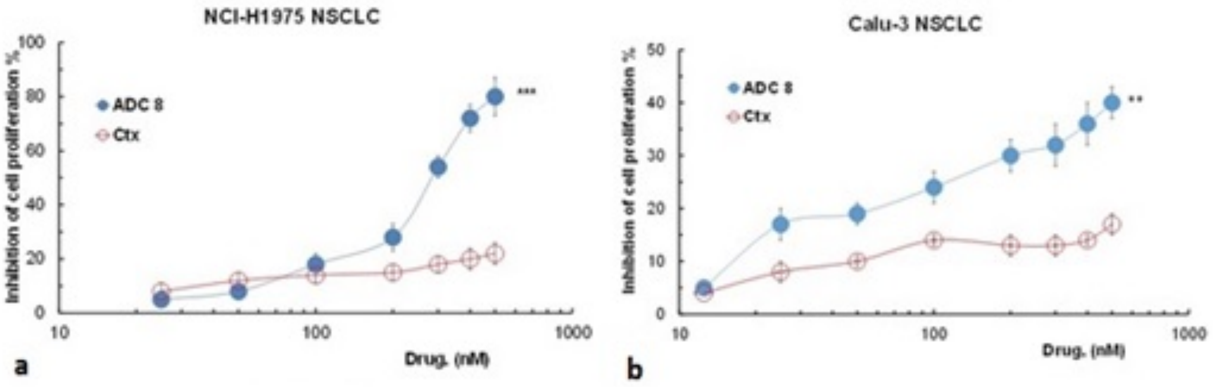

Figure S9: Antiproliferative activity of ADC **8** on NCI-H1975 and Calu-3 non-small cell lung carcinoma cells upon 6 days of treatment. Data are the mean (± SE) of percentage inhibition of two independent experiments. Mann-Whitney's test $^{**}p \leq 0.01$, $^{***}p \leq 0.00$

µM		Average area
1		12485,5
5		61835
10		677730,5
25		1015360,5
50		2358483

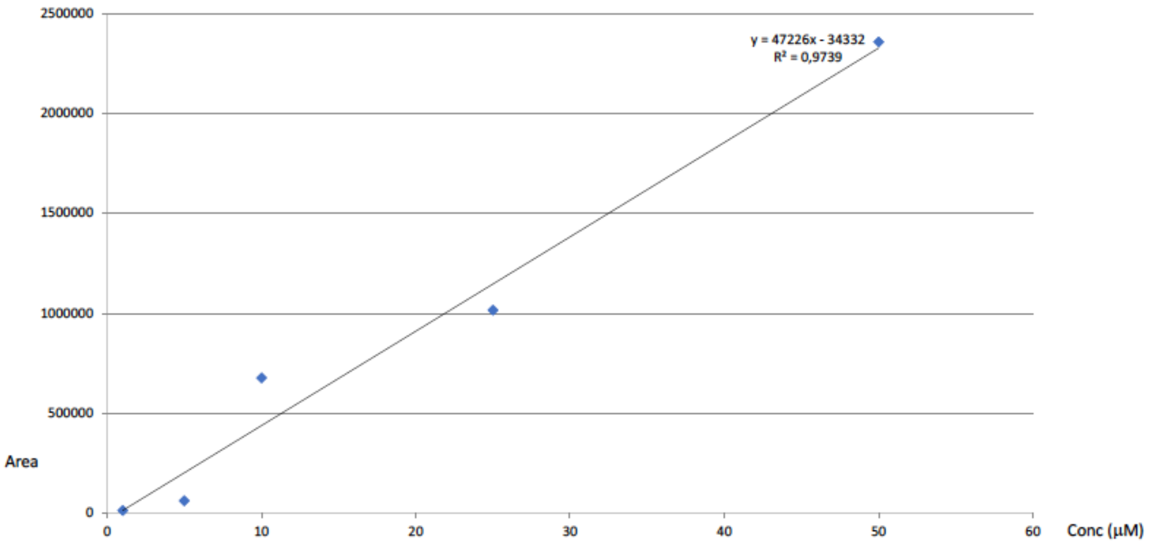

Fig S10a Calibration curve done at different concentrations of **2**. The peak monitored was 362.00

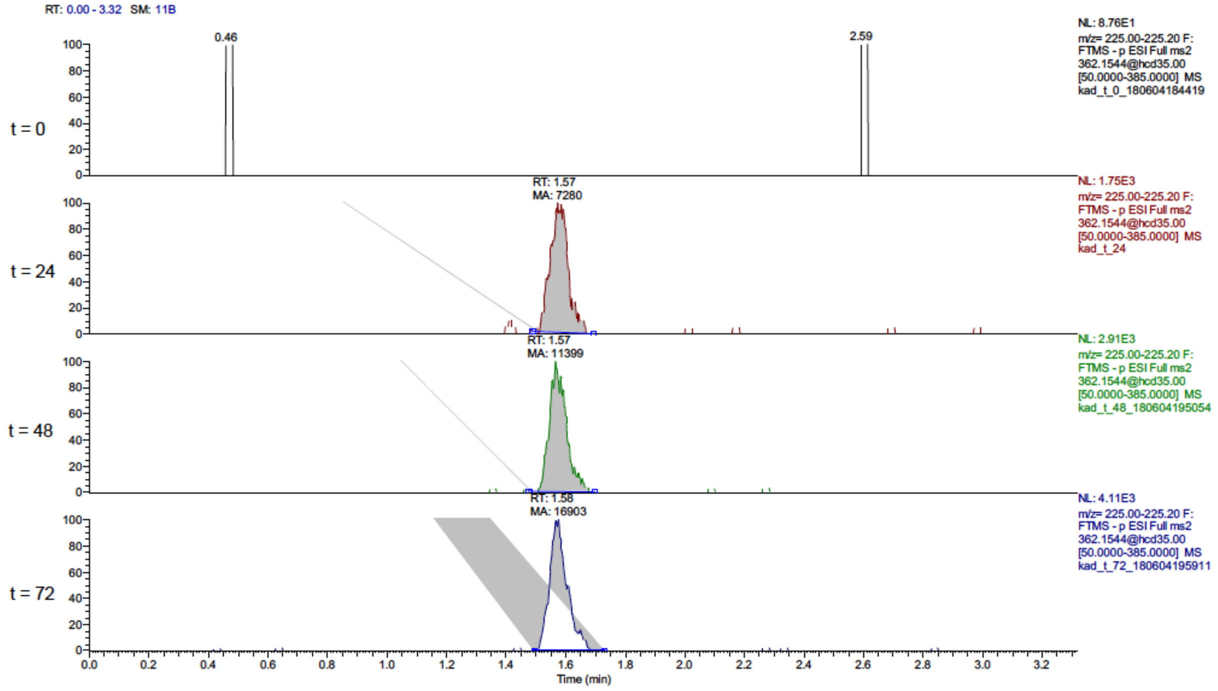

Fig S10b Time dependent formation of compound **2** from ADC **8** in the presence of human hepatic microsomes

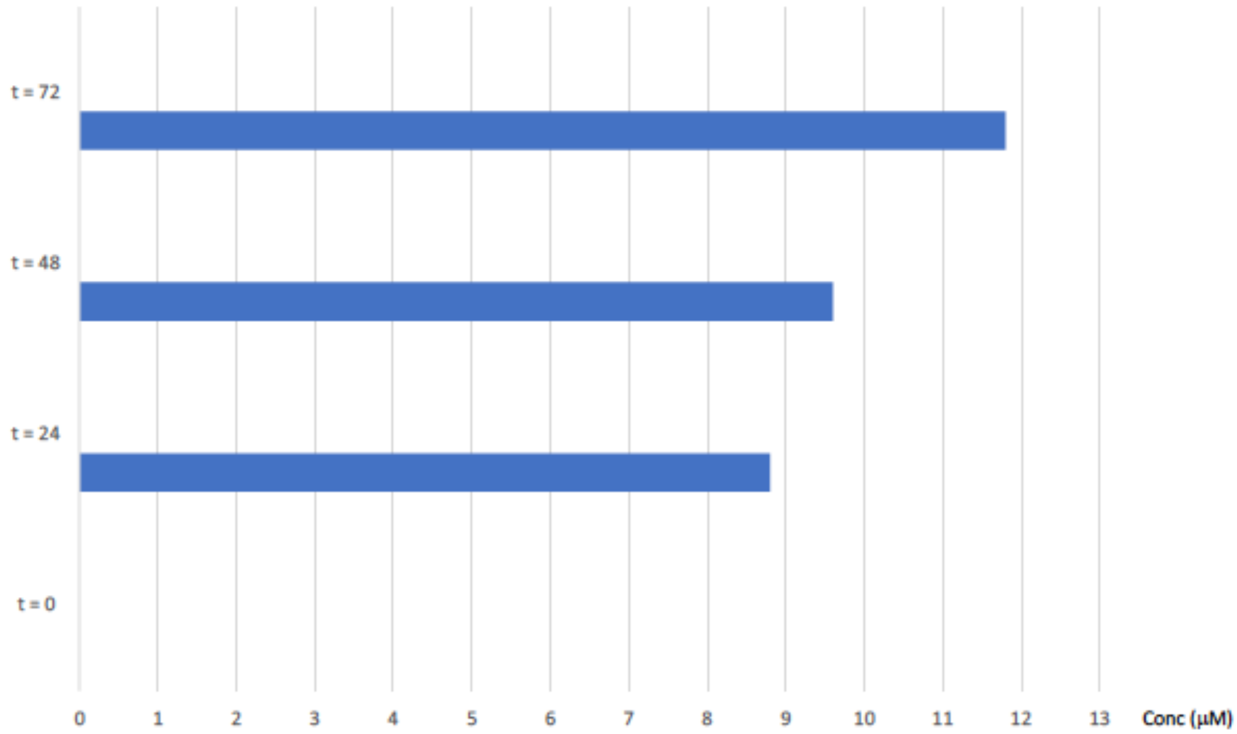

Fig S10c Time dependent increment of the concentration of compound **2** after hepatic microsome digestion of ADC **8**

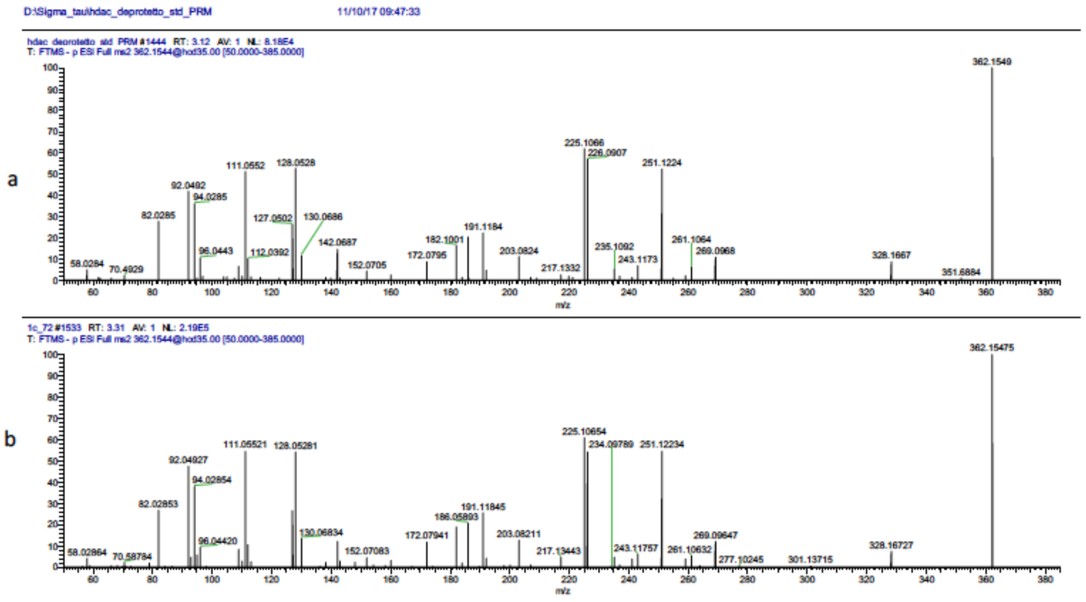

Fig S11 Comparison between the MS/MS spectra of (a) pure compound **2** and (b) the spectrum extracted from the HPLC/MS profiles in Fig. S10b

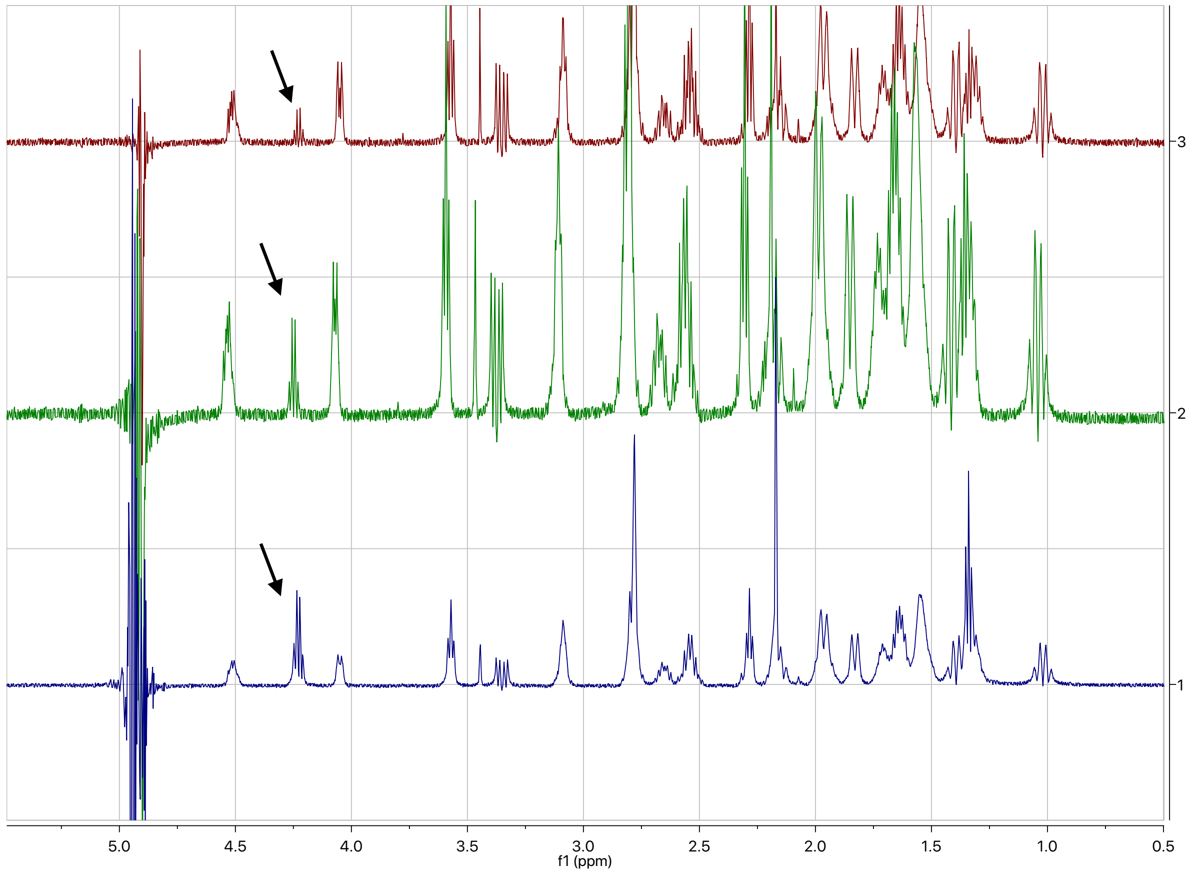

Fig S12. Comparison of ^{1}H 600 MHz spectra of compound **4** in PBS after 1.5 h (red line), 6 h (green line) and 12 h (blue line). Spectra recorded under H_2O signal suppression at 4.9 ppm. The arrow shows the onset of the signal relative to the ring opened succinimide acid **4a**.

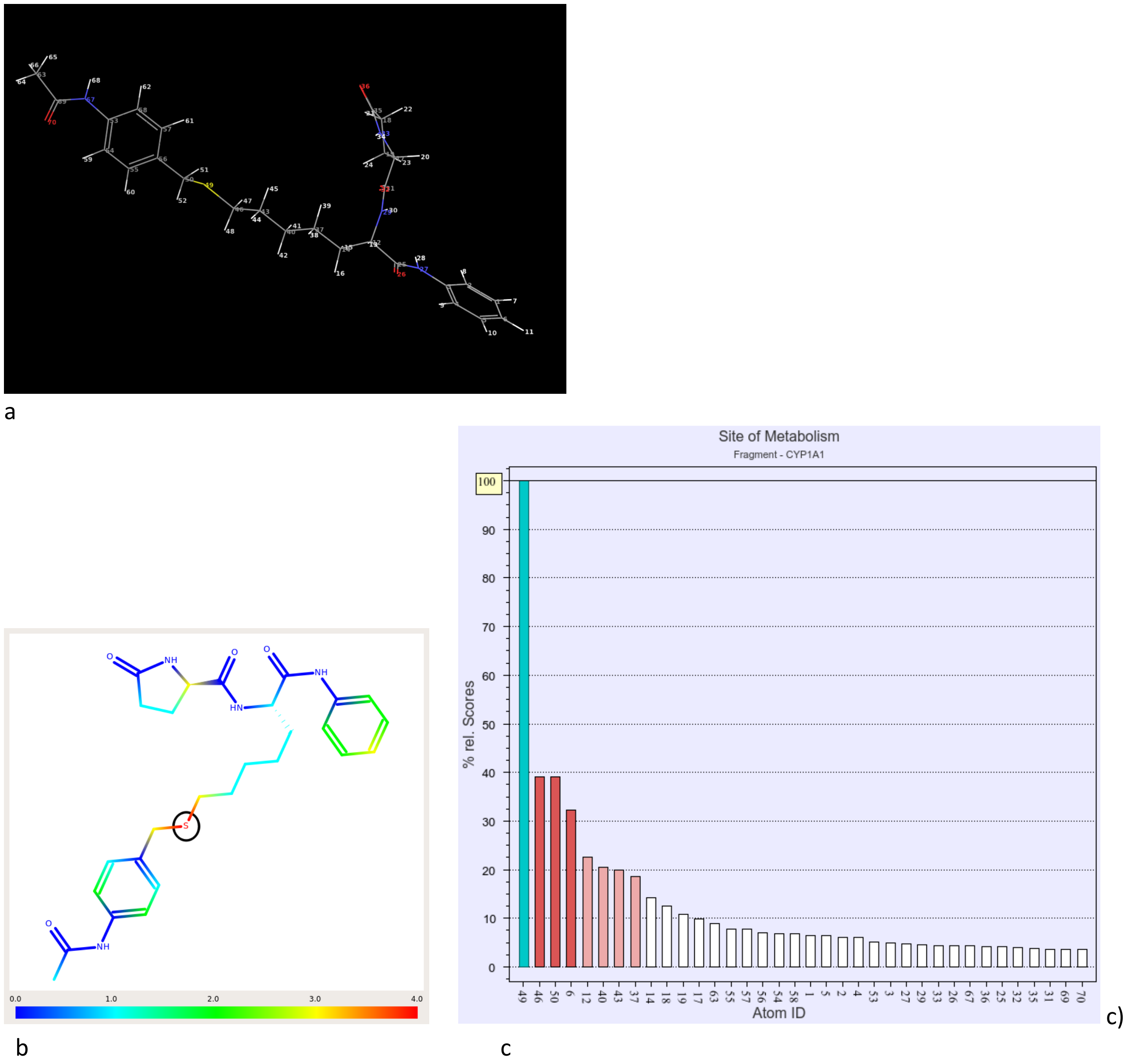

Fig S13. Prediction of oxidative metabolism. Software MetaSite from Molecular Discovery. a) Structure input derived from **7**. This compound has been used as a model for metabolites in silico prediction as the peptic linker is subjected to classical metabolism mediated by peptidases, and we are focusing our attention on cytochrome oxidative metabolism on the PAB-thioether moiety. The introduction of an acetamide system on the PAB-thioether doesn't impact in the reliability of the data obtained. b) Reactivity centers c) Reactivity score

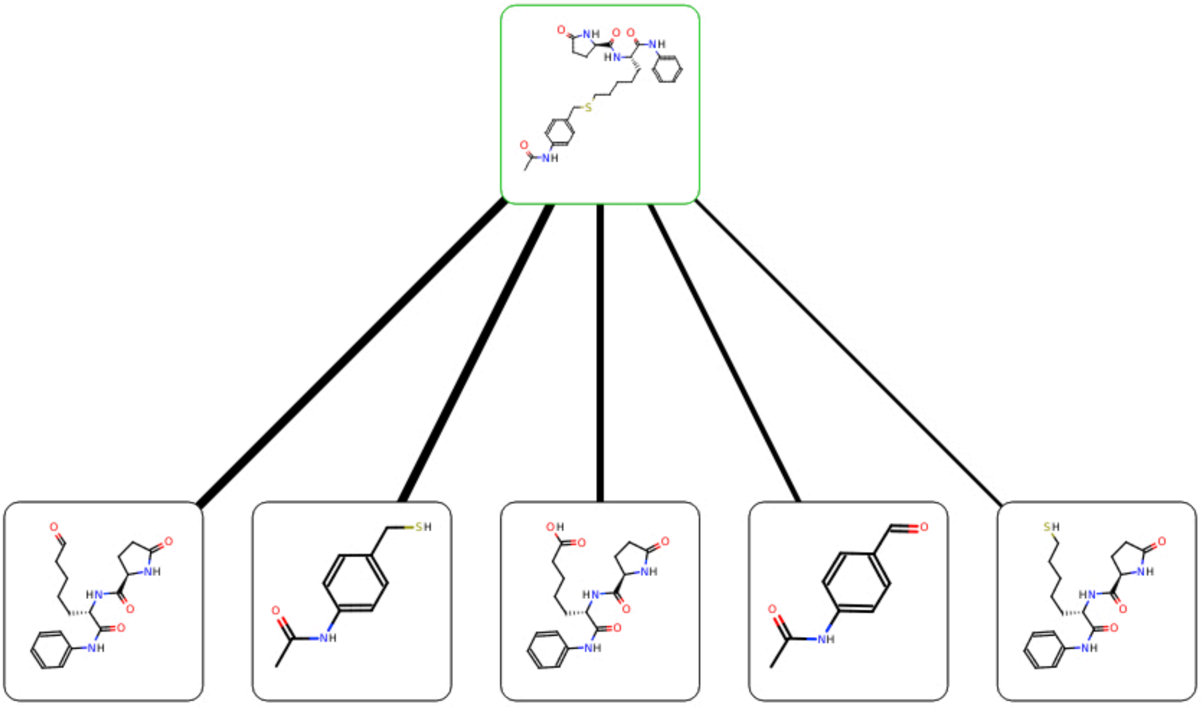

Fig S14 Tree of the most likely formed metabolites.

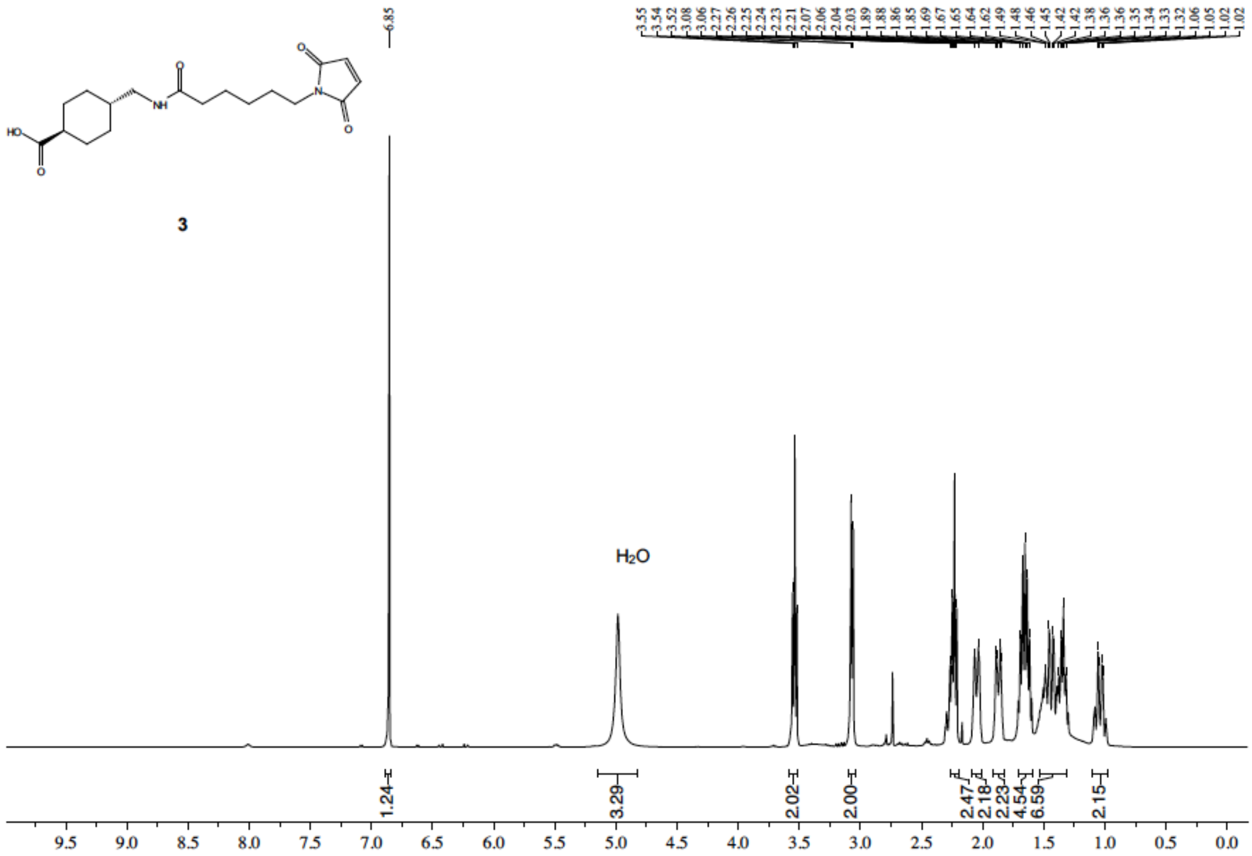
3.55 3.54 3.52 3.08 3.06 2.27 2.26 2.25 2.24 2.23 2.21 2.07 2.06 2.04 2.03 1.89 1.88 1.86 1.85 1.69 1.67 1.65 1.64 1.62 1.49 1.48 1.46 1.45 1.42 1.42 1.38 1.36 1.36 1.35 1.34 1.33 1.32 1.06 1.05 1.02 1.02
6.85
3
H_2O
1.24
3.29
2.02
2.00
2.47
2.18
2.23
4.54
6.59
2.15
9.5 9.0 8.5 8.0 7.5 7.0 6.5 6.0 5.5 5.0 4.5 4.0 3.5 3.0 2.5 2.0 1.5 1.0 0.5 0.0

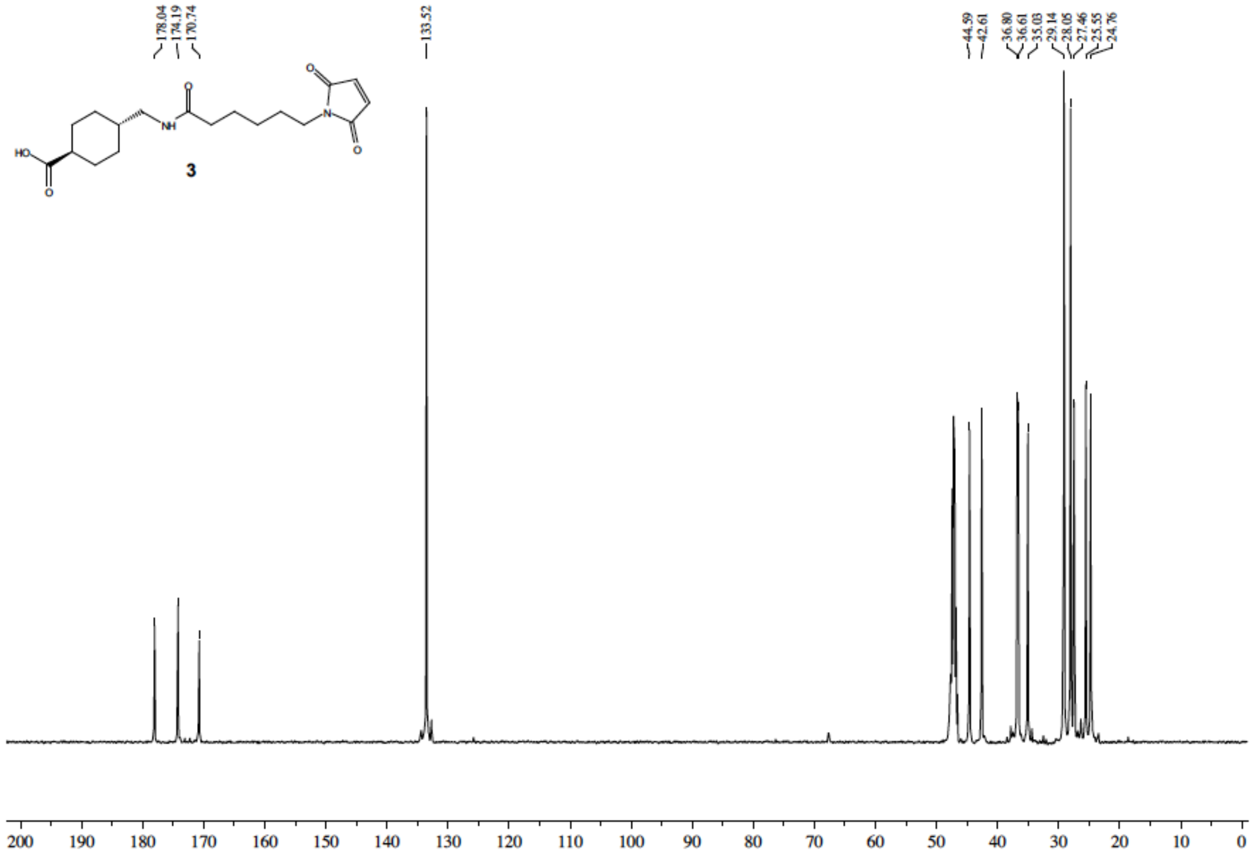
178.04 174.19 170.74
133.52
44.59 42.61 36.80 36.61 35.03 29.14 28.05 27.46 25.55 24.76
3
200 190 180 170 160 150 140 130 120 110 100 90 80 70 60 50 40 30 20 10 0

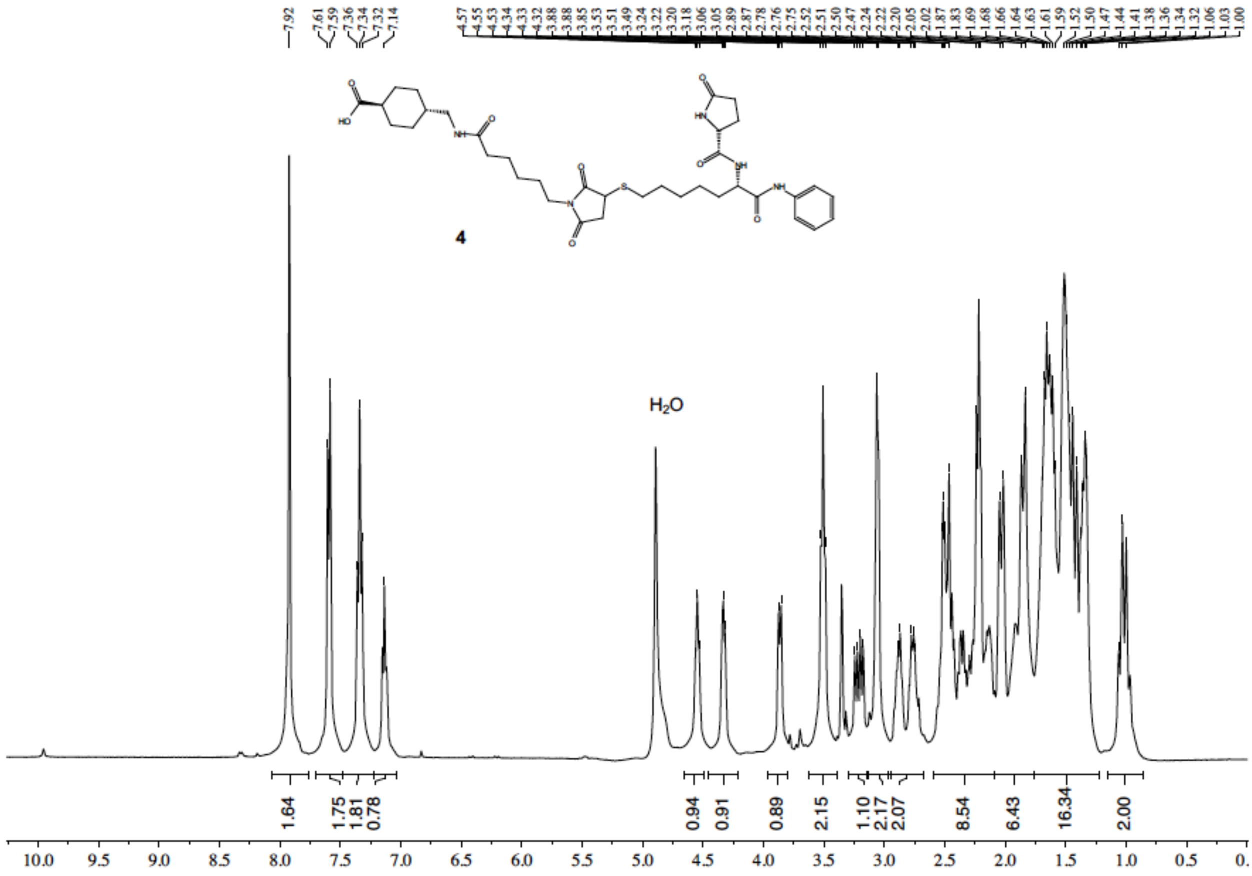

4
H2O
1.64
1.75
1.81
0.78
0.94
0.91
0.89
2.15
1.10
2.17
2.07
8.54
6.43
16.34
2.00
10.0 9.5 9.0 8.5 8.0 7.5 7.0 6.5 6.0 5.5 5.0 4.5 4.0 3.5 3.0 2.5 2.0 1.5 1.0 0.5

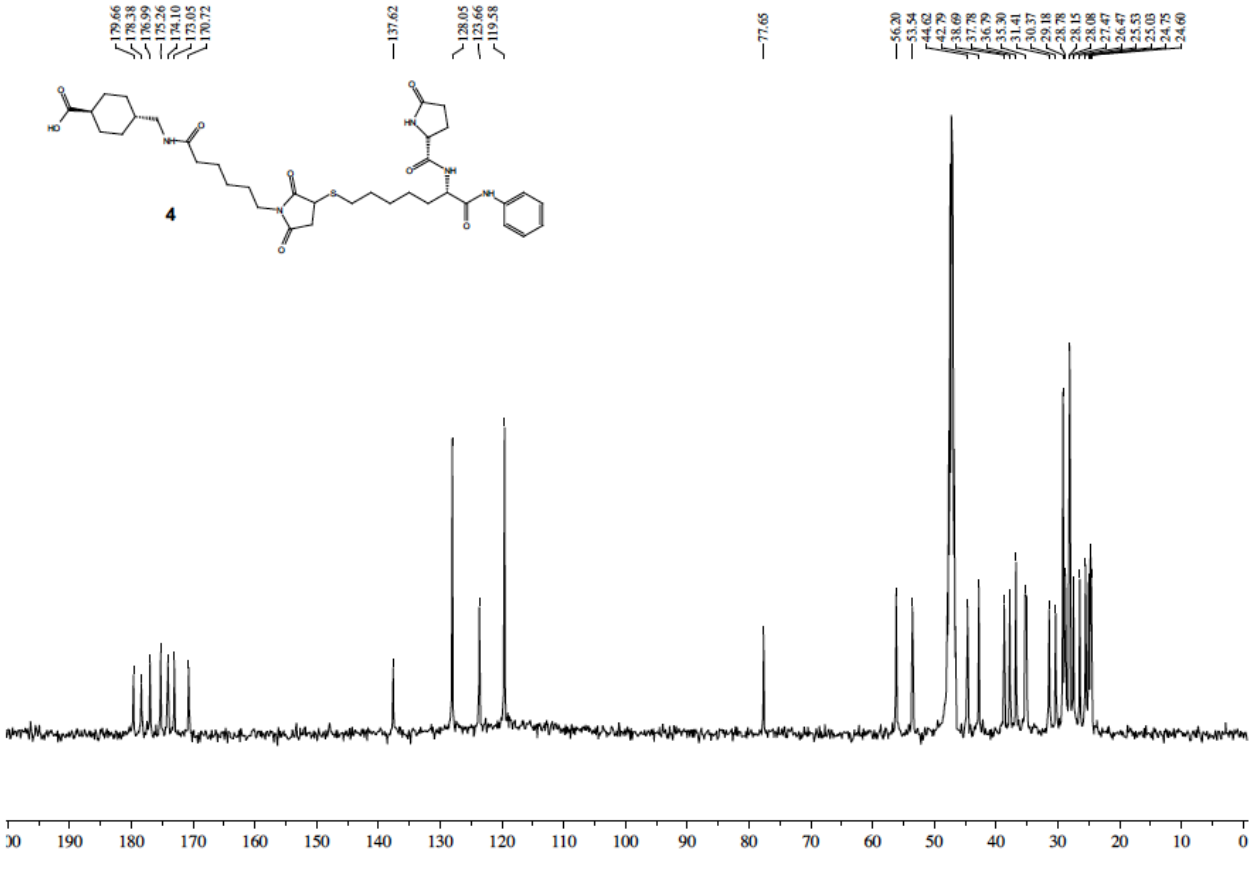

4
190 180 170 160 150 140 130 120 110 100 90 80 70 60 50 40 30 20 10 0

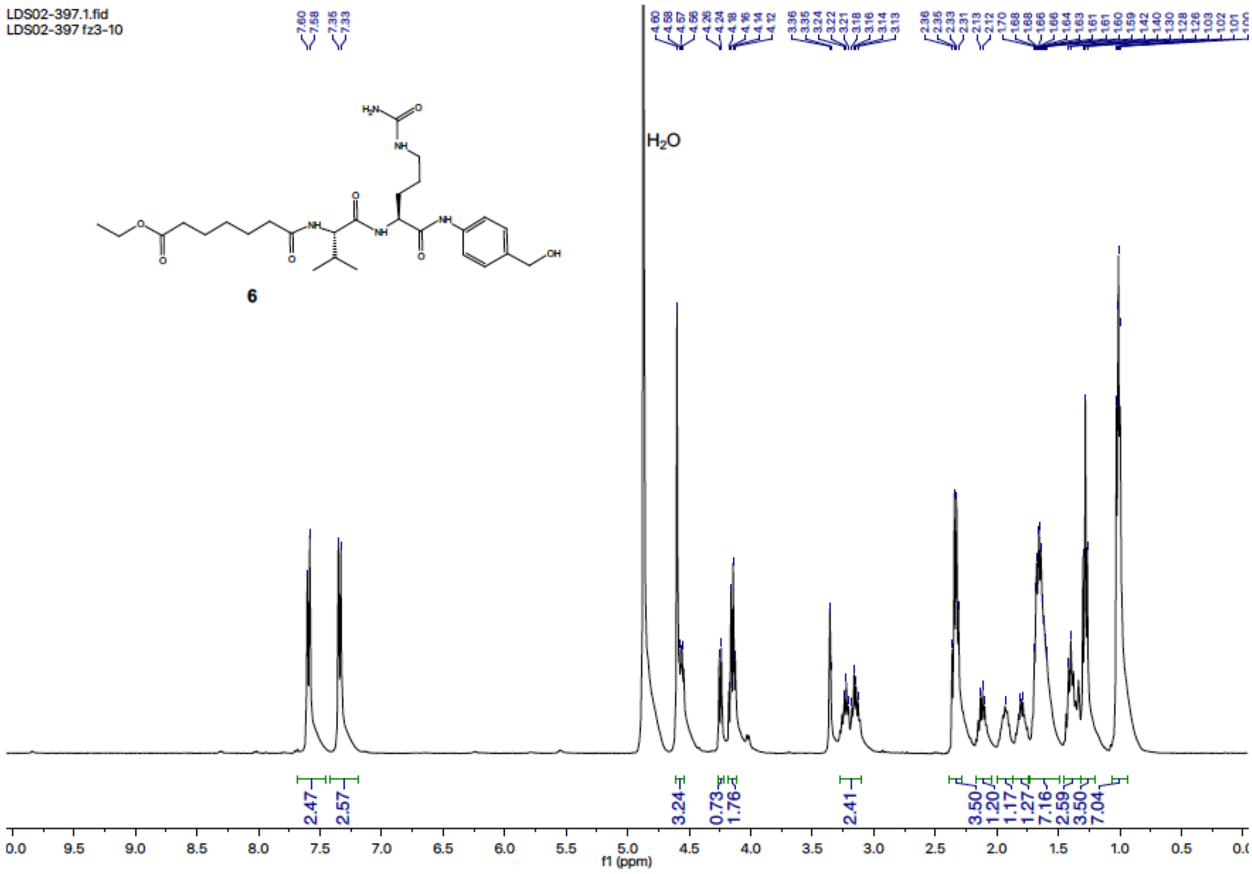
LDS02-397.1.fid
LDS02-397 fz3-10
H_2O
6
f1 (ppm)

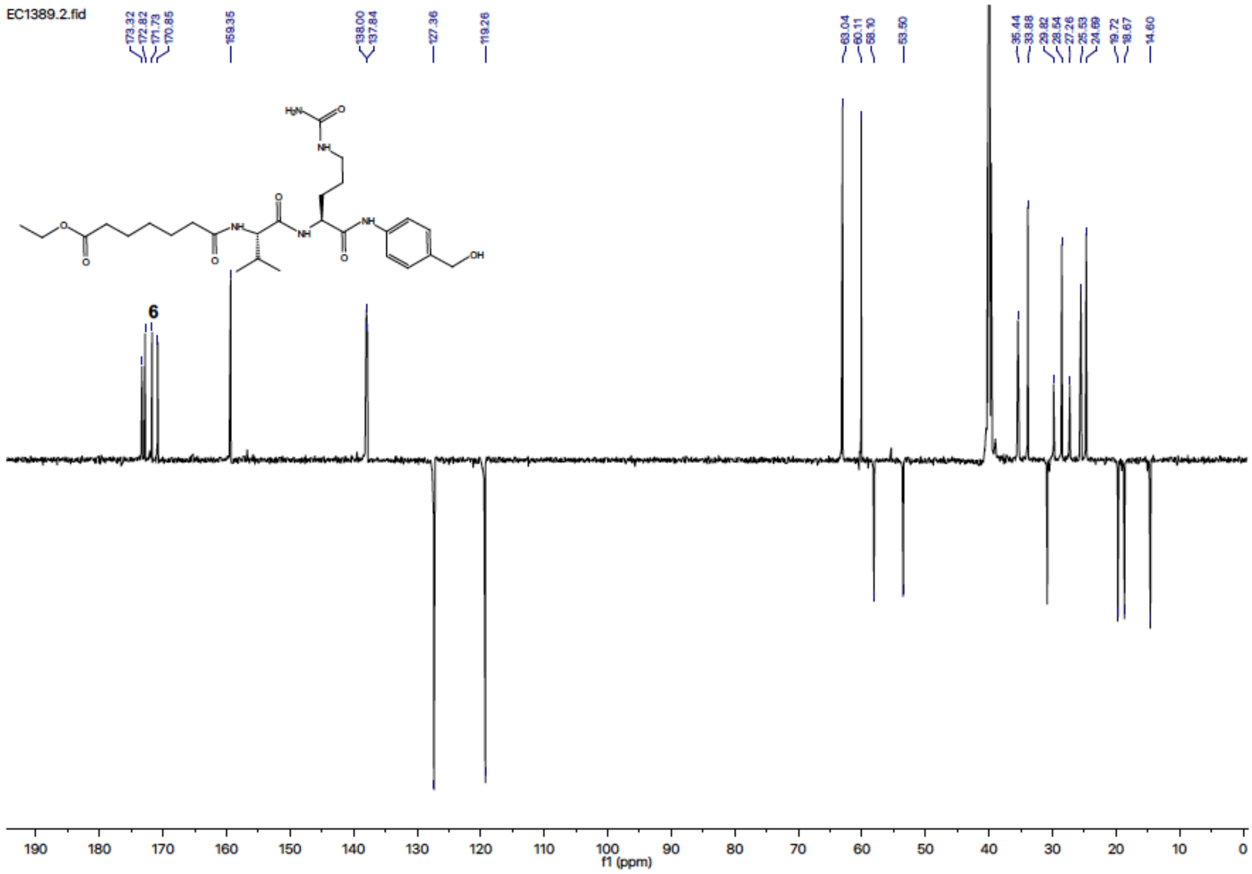
EC1389.2.fid
6
f1 (ppm)

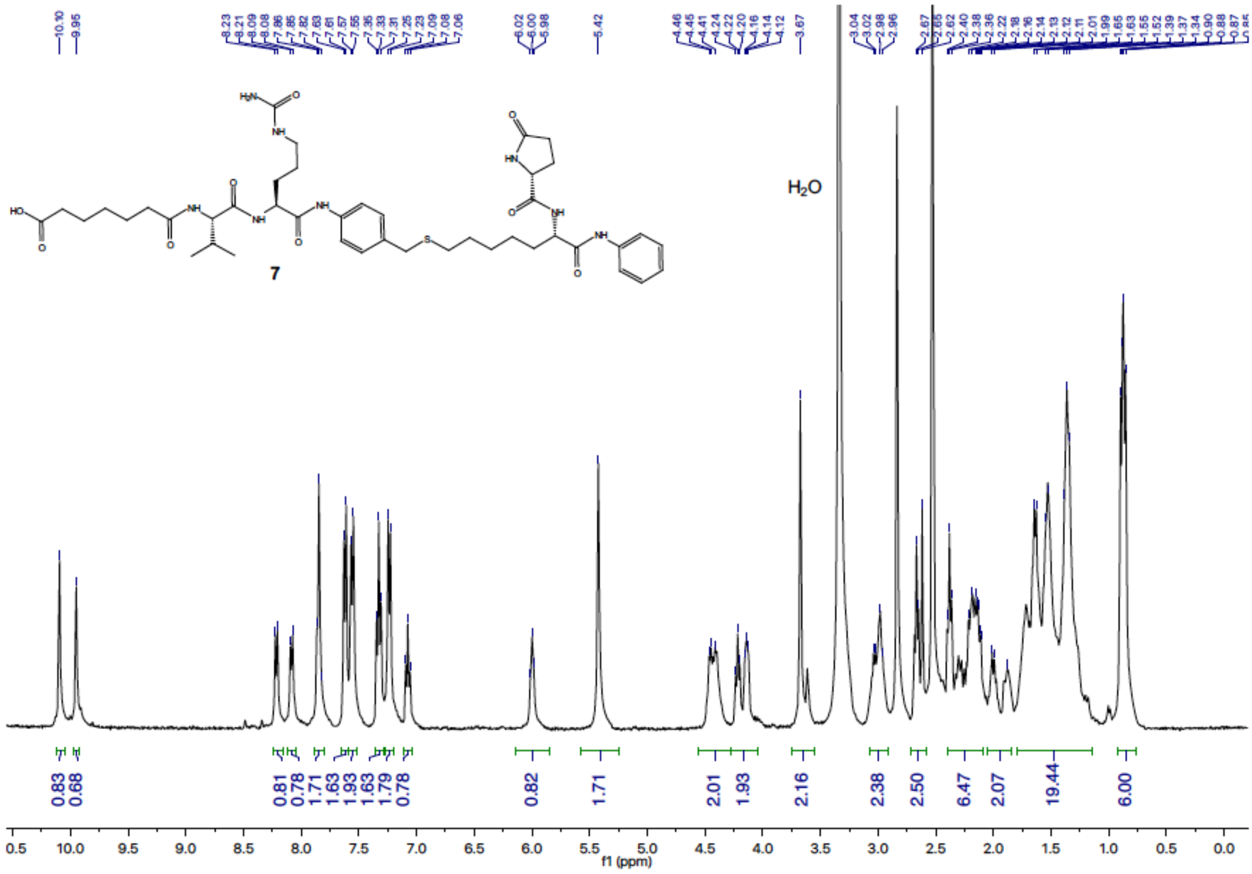

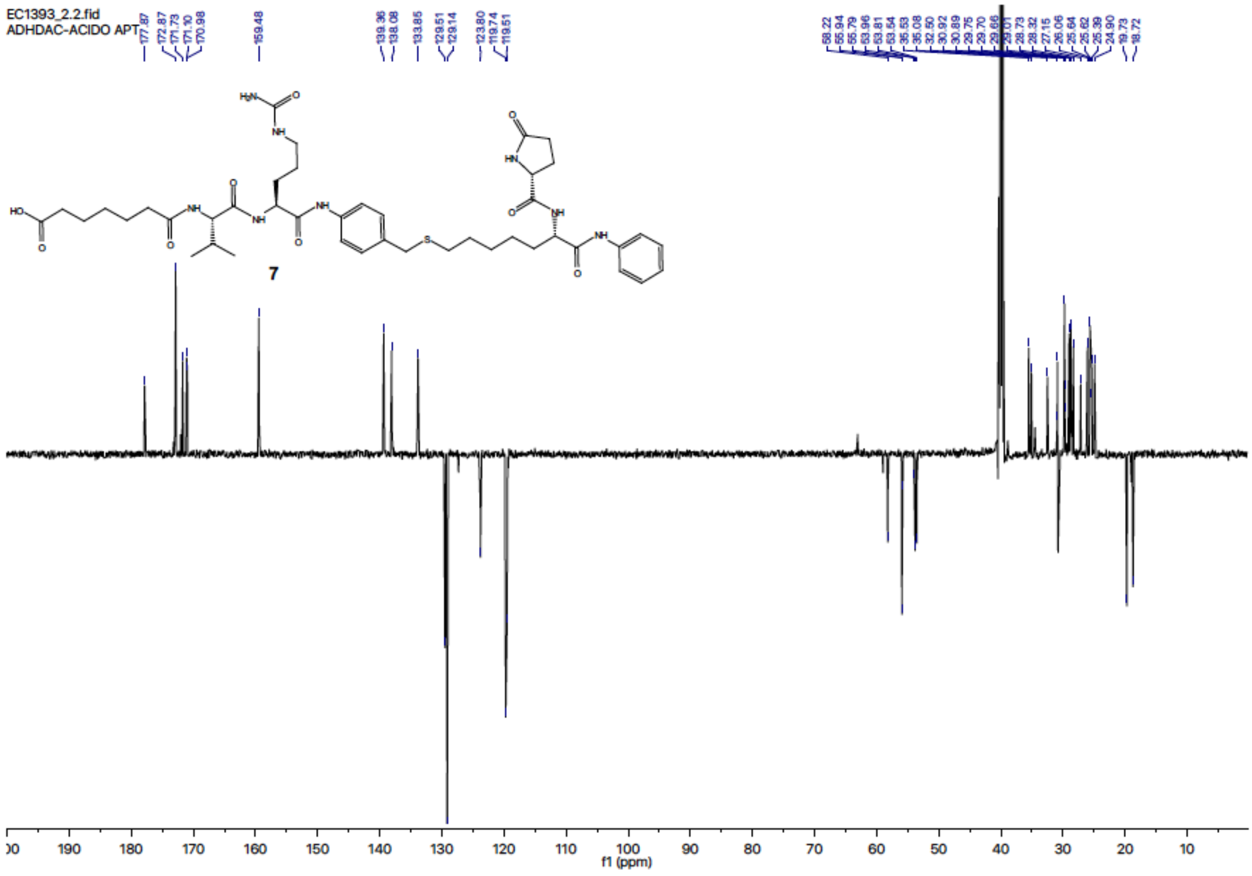

References

1 W. L. F. Armarego and D. D. (Douglas D. Perrin, *Purification of laboratory chemicals*, Butterworth Heinemann, 1997.

2 C. Kilkenny, W. J. Browne, I. C. Cuthill, M. Emerson and D. G. Altman, *Osteoarthr. Cartil.*, 2012, **20**, 256–260.

3 R. de Figueiredo, P. Oczipka, R. Fröhlich and M. Christmann, *Synthesis*, 2008, **2008**, 1316–1318.

4 H. Y. Song, M. H. Ngai, Z. Y. Song, P. A. MacAry, J. Hobley and M. J. Lear, *Org. Biomol. Chem.*, 2009, **7**, 3400.

5 G. M. Dubowchik, R. A. Firestone, L. Padilla, D. Willner, S. J. Hofstead, K. Mosure, J. O. Knipe, S. J. Lasch and P. A. Trail, *Bioconjug. Chem.*, 2002, **13**, 855–869.

6 A. J. Bessire, T. E. Ballard, M. Charati, J. Cohen, M. Green, M. H. Lam, F. Loganzo, B. Nolting, B. Pierce, S. Puthenveetil, L. Roberts, K. Schildknegt and C. Subramanyam, *Bioconjug. Chem.*, 2016, **27**, 1645–1654.

Complete author list for references with more than 10 authors:

1 W. L. F. Armarego and D. D. (Douglas D. Perrin, *Purification of laboratory chemicals*, Butterworth Heinemann, 1997.

2 C. Kilkenny, W. J. Browne, I. C. Cuthill, M. Emerson and D. G. Altman, *Osteoarthr. Cartil.*, 2012, **20**, 256–260.

3 R. de Figueiredo, P. Oczipka, R. Fröhlich and M. Christmann, *Synthesis (Stuttg).*, 2008, **2008**, 1316–1318.

4 H. Y. Song, M. H. Ngai, Z. Y. Song, P. A. MacAry, J. Hobley and M. J. Lear, *Org. Biomol. Chem.*, 2009, **7**, 3400.

5 G. M. Dubowchik, R. A. Firestone, L. Padilla, D. Willner, S. J. Hofstead, K. Mosure, J. O. Knipe, S. J. Lasch and P. A. Trail, *Bioconjug. Chem.*, 2002, **13**, 855–869.

6 A. J. Bessire, T. E. Ballard, M. Charati, J. Cohen, M. Green, M. H. Lam, F. Loganzo, B. Nolting, B. Pierce, S. Puthenveetil, L. Roberts, K. Schildknegt and C. Subramanyam, *Bioconjug. Chem.*, 2016, **27**, 1645–1654.
